# Effects of school neighborhood food environments on childhood obesity at multiple scales: a longitudinal kindergarten cohort study in the USA

**DOI:** 10.1186/s12916-019-1329-2

**Published:** 2019-05-22

**Authors:** Peng Jia, Hong Xue, Xi Cheng, Youfa Wang

**Affiliations:** 1 GeoHealth Initiative, Department of Earth Observation Science, Faculty of Geo-information Science and Earth Observation (ITC), University of Twente, 7500 Enschede, Netherlands; 2 International Initiative on Spatial Lifecourse Epidemiology (ISLE), Enschede, Netherlands; 3 Department of Health Behavior and Policy, School of Medicine, Virginia Commonwealth University, Richmond, VA 23298 USA; 4 Department of Geography, University at Buffalo, The State University of New York, Buffalo, NY 14261 USA; 5 Systems-Oriented Global Childhood Obesity Intervention Program, Fisher Institute of Health and Well-Being, College of Health, Ball State University, Muncie, IN 47306 USA; 6 Department of Nutrition and Health Sciences, College of Health, Ball State University, Muncie, IN 47306 USA

**Keywords:** Body mass index, Obesity, Overweight, Children, School neighborhood, Food environment, Obesogenic environment

## Abstract

**Background:**

School neighborhood food environment is recognized as an important contributor to childhood obesity; however, large-scale and longitudinal studies remain limited. This study aimed to examine this association and its variation across gender and urbanicity at multiple geographic scales.

**Methods:**

We used the US nationally representative Early Childhood Longitudinal Study–Kindergarten cohort data and included 7530 kindergarteners followed up from 1998 to 2007. The Census, road network, and Dun and Bradstreet commercial datasets were used to construct time-varying measurements of 11 types of food outlet within 800-m straight-line and road-network buffer zones of schools and school ZIP codes, including supermarket, convenience store, full-service restaurant, fast-food restaurant, retail bakery, dairy product store, health/dietetic food store, candy store, fruit/vegetable market, meat/fish market, and beverage store. Two-level mixed-effect and cluster-robust logistic regression models were performed to examine the association.

**Results:**

A higher body mass index (BMI) in 2007 was observed among children experiencing an increase of convenience stores in school neighborhoods during 1998–2007 (*β* = 0.39, *p* < 0.05), especially among girls (*β* = 0.50) and urban schoolchildren (*β* = 0.41), as well as among children with a decrease of dairy product stores (*β* = 0.39, *p* < 0.05), especially among boys (*β* = 1.86) and urban schoolchildren (*β* = 0.92). The higher obesity risk was associated with the increase of fast-food restaurants in urban schoolchildren (OR = 1.27 [95% CI = 1.02–1.59]) and of convenience stores in girls (OR = 1.41 [95% CI = 1.09–1.82]) and non-urban schoolchildren (OR = 1.60 [95% CI = 1.10–2.33]). The increase of full-service restaurants was related to lower obesity risk in boys (OR = 0.74 [95% CI = 0.57–0.95]). The decrease of dairy product stores was associated with the higher obesity risk (OR = 1.68 [95% CI = 1.07–2.65]), especially boys (OR = 2.92 [95% CI = 1.58–5.40]) and urban schoolchildren (OR = 1.67 [95% CI = 1.07–2.61]). The schoolchildren exposed to the decrease of meat/fish markets showed the lower obesity risk (OR = 0.57 [95% CI = 0.35–0.91]), especially urban schoolchildren (OR = 0.53 [95% CI = 0.32–0.87]). Results from analyses within 800-m straight-line buffer zones of schools were more consistent with our theory-based hypotheses than those from analyses within 800-m road-network buffer zones of schools and school ZIP codes.

**Conclusions:**

National data in the USA suggest that long-term exposure to the food environment around schools could affect childhood obesity risk; this association varied across gender and urbanicity. This study has important public health implications for future school-based dietary intervention design and urban planning.

**Electronic supplementary material:**

The online version of this article (10.1186/s12916-019-1329-2) contains supplementary material, which is available to authorized users.

## Background

Obesity prevalence has substantially increased among adolescents worldwide over the past 35 years [1–5]. Childhood obesity is associated with a number of serious health risks that can persist into adulthood [6]. Given the growing attention to the effects of food environments on childhood obesity, defined as “the availability, affordability, convenience, and desirability of various foods” surrounding individuals [7], a large number of studies have been undertaken to examine the relationships between residential food environment and adolescents’ weight status [8, 9]. For example, some cross-sectional studies showed that adolescents tended to have higher weight status if they had higher access to fast-food restaurants [10, 11] and convenience stores [12, 13] or lower access to grocery stores [14, 15], supermarkets [16–18], and full-service restaurants [10, 18].

School is a key site for effective obesity intervention and control [19], as adolescents spend a large portion of their day in school. Some previous studies found that the school neighborhood food environment was correlated with adolescents’ weight status at different levels [20–22]. For instance, the presence of a convenience store within an 800-m buffer of the school was associated with a higher overweight rate among the students [21]. However, nearly all existing evidence has been from cross-sectional and also local studies, which could lead to some mixed findings. For example, the presence of fast-food restaurants near schools was only associated with higher obesity risk at an individual level [20] instead of at a school level [21]. To the best knowledge of the authors, there have been few longitudinal, large-scale studies designed for examining the association between school neighborhood food environment and children’s weight status, with the dynamics between food environments and children’s weight status taken into consideration. In addition, previous studies have suggested that gender- and urbanicity-specific differences in the association may exist due to various dietary and physical activity patterns [19, 23–25]. This, however, was not found in school neighborhoods and also has not been examined in a longitudinal context. All these limitations warrant further research and investigation.

This study used nationally representative samples from the USA to examine (1) temporal changes of school neighborhood food environments between 1998 and 2007, (2) the longitudinal association between school neighborhood food environments and children’s weight status, and (3) the variation of the association across gender and urbanicity. Analyses were conducted at multiple geographic scales (800-m road-network buffer, 800-m straight-line buffer, and school ZIP code) to test the sensitivity of results and show the influence of choosing different geographic analysis units on the association. Findings of this study will deepen our understanding of how the structure of food environments in the vicinity of schools may affect children’s weight status, and help to inform future school-based childhood obesity interventions and urban planning in the USA and worldwide.

## Methods

### Study design and study sample

This study used the nationally representative data from the US Early Childhood Longitudinal Study–Kindergarten (ECLS-K) cohort, which was designed to examine child development under the sponsorship of several US federal government agencies, including the National Center for Education Statistics. The ECLS-K cohort includes about 22,000 kindergarteners from over 1200 schools in 1998–1999, and 9440 of them were successfully followed up until their 8th grades (2007) [26]. Data collected in 1998–1999 (baseline, hereafter referred to as the 1998 wave) and 2007 (the last follow-up survey) were analyzed. The study included only the students who had complete basic sociodemographic information and measured body mass index (BMI) and had attended the school in the contiguous USA (Alaska and Hawaii excluded) during 1998–2007.

### Outcome variables

Children’s body weight and height were measured twice during interviews, using a digital scale (Seca model 840, Seca North America West, Chino, CA) recording to the nearest 0.1 kg and a stadiometer (Shorr Productions LLC, Olney, MD) recording to the nearest 0.1 cm, respectively. The two weight measurements were averaged if they differed < 2.3 kg (i.e., 5 lbs), and the two height measurements were averaged if they differed < 5.08 cm (i.e., 2 in.) [27]. The *BMI* (in kg/m^2^) for each child was calculated by dividing body weight by squared height. *Obesity* was defined as gender-age-specific BMI ≥ 95th percentile of the 2000 CDC Growth Chart, while *overweight* (including obesity, hereafter called *overweight*/*obesity*) was ≥ 85th percentile [28].

### Exposure variables

The Dun and Bradstreet (D&B) commercial datasets in 1998 and 2007, including all food outlets (points of interest) with geographic coordinates, were used to characterize the food environment surrounding the schools that ECLS-K children attended in 1998 and 2007. According to the hierarchical Standard Industrial Classification (SIC) codes (Additional file 1: Table S1), 11 categories of food outlets were extracted from D&B datasets and included in the analyses: supermarket, convenience store, full-service restaurant (eating places excluding fast-food restaurants/stands), fast-food restaurant/stand, retail bakery, dairy product store, health/dietetic food store, candy/nut/confectionery store, fruit/vegetable market, meat/fish market, and beverage store.

The *density of each category of food outlets* (per km^2^) in 1998 and 2007 was separately calculated within three types of geographic units in which food environments have been associated with child weight status [29]: (1) 800-m straight-line buffer, by creating a circular buffer zone with a radius of 800 m centered on each school; (2) 800-m road-network buffer, where it covers 800 m to travel from any point on the boundary of the buffer zone (normally in an irregular shape) to the school along the shortest path on the basis of national road network datasets; and (3) school ZIP code, extracted from the year 2000 US ZIP code boundaries. The changes in each variable from 1998 to 2007 were calculated by subtracting the density in 1998 from the density in 2007 in the school neighborhood, with each sample labeled as one of the three categories for each variable: *decreased* (negative change), *constant* (no change), and *increased* (positive change). After a data screening, several categories of food outlet around many schools were sparse or absent. For more meaningful analyses and interpretation of model coefficients, as well as an easy comparison among the three scales of analyses, all samples were divided into *absence* (density = 0) and *presence* (density > 0) groups based on the density of each type of food outlet.

Considering the degree of healthiness of the food available in each type of food outlets, we hypothesized that the increased exposure to supermarket, full-service restaurant, health/dietetic food store, fruit/vegetable market, and beverage store was associated with lower weight status, while the increased exposure to convenience store, fast-food restaurant, retail bakery, dairy product store, candy store, and meat/fish market was associated with higher weight status.

### Covariates

Child-level covariates included age, gender, race/ethnicity (white, black, Hispanic, Asian, and others), socioeconomic status (SES), parental education, and urbanicity. Children’s SES was delineated into four categories based on their parents’ report of their household annual income: ≤ $30,000, $30,000–50,000, $50,000–75,000, and > $75,000. Parental education was determined based on the parent who had the higher education level, recoded as four categories: high school and below, vocational/tech/college, bachelor’s degree, and graduate degree. The urbanicity of the school’s location was either urban (urbanized areas of ≥ 50,000 inhabitants) or non-urban (small towns of < 50,000 inhabitants and rural).

### Statistical analysis

First, *χ*^2^ tests (for categorical variables) and *t* tests (for continuous variables) were conducted to identify significant disparities in children’s sociodemographic characteristics and weight status and the food environment in school neighborhoods between genders. McNemar’s tests (for categorical variables) and paired *t* tests (for continuous variables) were used to examine the significance of the changes in child weight status and food environments during 1998–2007.

Given the nested data structure (i.e., children within schools), two-level mixed-effect and cluster-robust logistic regression models were performed to estimate the associations of the changes in school neighborhood food environments during 1998–2007 with children’s BMI and weight status (i.e., overweight/obesity and obesity only) in 2007, respectively. The school-level random intercept represented the variation of BMI (or weight status) among children across schools. All models were adjusted for children’s baseline age, gender, race/ethnicity, SES, parental education, urbanicity, BMI (or weight status), and exposures to school neighborhood food environments. We also fitted separate models to examine the potential variation in these associations by gender and urbanicity.

All spatial operations and analyses were conducted in ArcGIS (Version 10.4.1, Esri, Redlands, CA), including the construction of food environment measures at different scales in Geographic Information Systems (GIS). All statistical analyses were performed using Stata 14 (College Station, TX), with sampling weight and complex survey design incorporated to compensate for non-response (loss to follow-up) and unequal probabilities of selection at each sampling stage [9, 30, 31]. In the main text, we showed the results from the analyses conducted within 800-m straight-line buffer zones, compared these results with those from the analyses conducted within 800-m road-network buffer zones and school ZIP codes (Additional file 1), and discussed all the results.

## Results

### Sample characteristics

Our final analytical sample included 7530 children, which had no significant differences from those excluded ones in terms of the distribution of children’s sociodemographic characteristics and weight status (Additional file 1: Table S2). At baseline in 1998, the mean age of the included children was 6.2 years, with boys slightly older than girls (*p* = 0.012) (Table 1). The baseline weight status was similar between genders, with a mean BMI of 16.4 kg/m^2^ and the prevalence of overweight/obesity and obesity being 26.6% and 11.3%, respectively. The significant increases that occurred during 1998–2007 in mean BMI (from 16.4 to 22.9, *p* < 0.001) and prevalence of overweight/obesity (from 26.6% to 35.5%, *p* < 0.001) and obesity (from 11.3% to 19.5%, *p* < 0.001) also occurred in boys and girls separately. In 2007, girls had developed a significantly higher BMI than boys on average (23.2 vs. 22.6, *p* = 0.013), but boys had a marginally higher prevalence of obesity than girls (21.0% vs. 18.0%, *p* = 0.062).

**Table 1:** Children’s sociodemographic characteristics at baseline and weight status of the US children at baseline and fifth wave of ECLS-K

Variables	Percent or mean ± SD			*p* value^a^
	All (*n* = 7530)	Boys (*n* = 3780)	Girls (*n* = 3750)	
Sociodemographic characteristics (1998)				
Age (years)	6.2 ± 0.4	6.3 ± 0.3	6.2 ± 0.3	*0.012*
Race/ethnicity				0.153
White	58.0	58.5	57.5	
Black	17.5	18.0	17.0	
Hispanic	18.2	18.4	18.1	
Asian	2.9	2.1	3.7	
Others	3.4	3.0	3.7	
Parental education				0.227
≤ High school	33.8	35.5	32.1	
Vocational/college	32.5	31.9	33.1	
Bachelor	19.2	19.1	19.3	
≥ Graduate	14.5	13.5	15.5	
Household annual income ($)				0.503
≤ 30,000	35.0	35.7	34.2	
> 30,000 but ≤ 50,000	24.1	24.4	23.8	
> 50,000 but ≤ 75,000	18.4	17.3	19.6	
> 75,000	22.5	22.6	22.4	
Urbanicity				0.797
Urban	68.4	68.1	68.6	
Non-urban	31.6	31.9	31.4	
Weight status^b^ (1998)				
BMI (kg/m^2^)	16.4 ± 2.2	16.4 ± 2.0	16.4 ± 2.3	0.684
Overweight and obesity	26.6	25.9	27.3	0.435
Obesity	11.3	11.6	10.9	0.590
Weight status^b^ (2007)				
BMI (kg/m^2^)	22.9 ± 5.7	22.6 ± 5.2	23.2 ± 5.8	*0.013*
Overweight and obesity	35.5	35.6	35.4	0.903
Obesity	19.5	21.0	18.0	0.062

Sampling weights were used in the analyses

^a^*p* values tested the differences in each variable between genders and were based on *χ*^2^ tests for categorical variables or *t* tests for continuous variables

^b^Children were classified as overweight and obesity if their sex-age-specific body mass index (BMI) ≥ 85th and 95th percentiles of the 2000 CDC Growth Chart, respectively

Italicized numbers indicate statistical significance *p* < 0.05

During 1998–2007, the numbers of food outlets in all types within school buffer zones had increased (*p* < 0.05). Full-service restaurants, supermarkets, and fast-food restaurants increased more than other types of food outlet. No gender differences were found in the exposure to any type of food outlet both in 1998 and 2007 (Table 2).

**Table 2:** School neighborhood food environments surrounding US children at baseline and fifth wave of ECLS-K

Food outlet type	Percent or mean ± SD			*p* value^a^
	All (*n* = 7530)	Boys (*n* = 3780)	Girls (*n* = 3750)	
Supermarket				
1998	0.56 ± 2.02	0.51 ± 1.77	0.62 ± 2.13	0.263
2007	0.83 ± 3.63	0.76 ± 3.39	0.91 ± 3.63	0.429
1998–2007				0.206
Decreased	16.1	15.3	17.0	
Constant	13.3	12.3	14.2	
Increased	70.6	72.4	68.8	
Convenience store				
1998	0.13 ± 0.25	0.13 ± 0.22	0.14 ± 0.26	0.460
2007	0.19 ± 0.40	0.19 ± 0.35	0.20 ± 0.41	0.456
1998–2007				0.937
Decreased	16.7	16.5	17.0	
Constant	19.4	19.6	19.2	
Increased	63.9	63.9	63.8	
Full-service restaurant				
1998	1.38 ± 5.14	1.28 ± 4.57	1.48 ± 5.36	0.163
2007	1.81 ± 6.93	1.72 ± 6.10	1.90 ± 7.27	0.371
1998–2007				0.278
Decreased	8.0	8.2	7.8	
Constant	5.9	5.2	6.6	
Increased	86.1	86.6	85.6	
Fast-food restaurant				
1998	0.25 ± 0.48	0.24 ± 0.43	0.26 ± 0.51	0.234
2007	0.43 ± 0.97	0.42 ± 0.87	0.44 ± 0.99	0.527
1998–2007				0.295
Decreased	3.8	4.0	3.5	
Constant	11.3	10.3	12.3	
Increased	84.9	85.7	84.2	
Retail bakery				
1998	0.16 ± 0.50	0.14 ± 0.43	0.17 ± 0.53	0.103
2007	0.20 ± 0.72	0.19 ± 0.59	0.22 ± 0.80	0.141
1998–2007				0.818
Decreased	16.0	16.3	15.8	
Constant	29.2	28.6	29.7	
Increased	54.8	55.1	54.5	
Dairy product store				
1998	0.05 ± 0.13	0.05 ± 0.12	0.05 ± 0.13	0.815
2007	0.08 ± 0.20	0.08 ± 0.16	0.08 ± 0.21	0.369
1998–2007				0.194
Decreased	5.9	6.1	5.6	
Constant	31.3	29.7	32.9	
Increased	62.8	64.2	61.5	
Health food store				
1998	0.07 ± 0.26	0.07 ± 0.25	0.07 ± 0.26	0.560
2007	0.10 ± 0.40	0.10 ± 0.35	0.11 ± 0.43	0.649
1998–2007				0.320
Decreased	12.3	11.8	12.7	
Constant	35.5	34.4	36.7	
Increased	52.2	53.8	50.6	
Candy store				
1998	0.04 ± 0.30	0.03 ± 0.24	0.05 ± 0.34	0.098
2007	0.04 ± 0.28	0.03 ± 0.22	0.05 ± 0.32	0.100
1998–2007				0.166
Decreased	13.8	14.7	12.8	
Constant	54.8	55.6	54.1	
Increased	31.4	29.7	33.1	
Fruit/vegetable market				
1998	0.03 ± 0.22	0.03 ± 0.18	0.04 ± 0.25	0.155
2007	0.05 ± 0.27	0.04 ± 0.20	0.06 ± 0.31	0.090
1998–2007				0.305
Decreased	5.9	6.3	5.4	
Constant	65.9	66.7	65.2	
Increased	28.2	27.0	29.4	
Meat/fish market				
1998	0.08 ± 0.40	0.07 ± 0.35	0.09 ± 0.43	0.230
2007	0.09 ± 0.45	0.08 ± 0.35	0.11 ± 0.51	0.207
1998–2007				0.509
Decreased	12.0	12.4	11.6	
Constant	52.3	51.0	53.6	
Increased	35.7	36.6	34.8	
Beverage store				
1998	0.05 ± 0.21	0.05 ± 0.21	0.05 ± 0.21	0.768
2007	0.11 ± 0.44	0.11 ± 0.45	0.11 ± 0.38	0.978
1998–2007				0.861
Decreased	4.7	5.0	4.5	
Constant	33.7	33.5	33.8	
Increased	61.6	61.5	61.7	

Sampling weights were used in the analyses

^a^*p* values tested the differences in each variable between genders and were based on *χ*^2^ tests for categorical variables or *t* tests for continuous variables

### Associations of school neighborhood food environments and child BMI

A higher BMI was observed among the children who attended schools with an increase of convenience stores in neighborhoods during 1998–2007 (*β* = 0.39, *p* < 0.05), especially among girls (*β* = 0.50, *p* < 0.05) and urban schoolchildren (*β* = 0.41, *p* < 0.05), compared to their counterparts who experienced a constant density of convenience stores in their school neighborhoods over the 9-year period (Table 3). The children who attended schools with a decrease of dairy product stores in neighborhoods also showed a higher BMI (*β* = 0.86, *p* < 0.05), especially boys (*β* = 1.86, *p* < 0.001) and urban schoolchildren (*β* = 0.92, *p* < 0.05). These effects of dairy product stores on child BMI were consistent with the results from the analyses within 800-m road-network buffer zones (Additional file 1: Table S3).

**Table 3:** Associations (coefficient and standard error) of changes in school neighborhood food environments within 800-m straight-line buffer zones during 1998–2007 with child body mass index in 2007

Food outlet type	All (*n* = 7530)		Boys (*n* = 3780)		Girls (*n* = 3750)		Urban (*n* = 5040)		Non-urban (*n* = 2490)	
Supermarket										
1998 (/km^2^)										
0 (ref)										
> 0	− 0.21	(0.19)	− 0.08	(0.22)	− 0.26	(0.27)	− 0.27	(0.23)	− 0.15	(0.38)
1998–2007										
Decreased	− 0.08	(0.23)	− 0.12	(0.26)	− 0.07	(0.34)	0.02	(0.27)	− 0.07	(0.48)
Constant (ref)										
Increased	0.02	(0.18)	0.03	(0.21)	− 0.04	(0.25)	0.14	(0.20)	− 0.35	(0.39)
Convenience store										
1998 (/km^2^)										
0 (ref)										
> 0	− 0.05	(0.19)	− 0.08	(0.22)	− 0.07	(0.27)	− 0.19	(0.22)	0.18	(0.42)
1998–2007										
Decreased	0.21	(0.25)	0.27	(0.28)	0.21	(0.36)	0.37	(0.30)	− 0.05	(0.47)
Constant (ref)										
Increased	0.39*	(0.17)	0.34	(0.20)	0.50*	(0.25)	0.41*	(0.21)	0.39	(0.36)
Full-service restaurant										
1998 (/km^2^)										
0 (ref)										
> 0	− 0.06	(0.20)	− 0.07	(0.23)	− 0.10	(0.29)	0.09	(0.25)	− 0.30	(0.38)
1998–2007										
Decreased	0.02	(0.24)	− 0.19	(0.28)	0.33	(0.35)	− 0.10	(0.30)	0.12	(0.45)
Constant (ref)										
Increased	− 0.06	(0.18)	− 0.39	(0.21)	0.36	(0.26)	− 0.13	(0.23)	− 0.03	(0.33)
Fast-food restaurant										
1998 (/km^2^)										
0 (ref)										
> 0	− 0.07	(0.20)	0.05	(0.23)	− 0.26	(0.28)	− 0.09	(0.23)	− 0.11	(0.43)
1998–2007										
Decreased	0.31	(0.27)	0.38	(0.31)	0.30	(0.39)	0.20	(0.30)	0.72	(0.67)
Constant (ref)										
Increased	0.28	(0.18)	0.10	(0.20)	0.44	(0.26)	0.33	(0.20)	0.34	(0.41)
Retail bakery										
1998 (/km^2^)										
0 (ref)										
> 0	− 0.07	(0.22)	0.04	(0.26)	− 0.15	(0.32)	− 0.21	(0.25)	0.54	(0.63)
1998–2007										
Decreased	− 0.43	(0.29)	− 0.43	(0.34)	− 0.48	(0.42)	− 0.32	(0.32)	− 0.58	(0.79)
Constant (ref)										
Increased	− 0.10	(0.20)	0.13	(0.24)	− 0.46	(0.29)	− 0.05	(0.22)	0.00	(0.60)
Dairy product store										
1998 (/km^2^)										
0 (ref)										
> 0	− 0.26	(0.29)	− 0.58	(0.33)	0.04	(0.41)	− 0.15	(0.31)	− 1.30	(0.89)
1998–2007										
Decreased	0.86*	(0.39)	1.86***	(0.45)	− 0.24	(0.57)	0.92*	(0.42)	0.64	(1.40)
Constant (ref)										
Increased	− 0.11	(0.21)	0.27	(0.24)	− 0.56	(0.30)	− 0.07	(0.23)	− 0.37	(0.50)
Health food store										
1998 (/km^2^)										
0 (ref)										
> 0	− 0.10	(0.28)	− 0.18	(0.32)	− 0.00	(0.41)	− 0.14	(0.31)	0.34	(0.79)
1998–2007										
Decreased	0.14	(0.36)	0.04	(0.42)	0.22	(0.52)	0.11	(0.39)	0.03	(1.17)
Constant (ref)										
Increased	− 0.06	(0.22)	− 0.16	(0.26)	0.09	(0.31)	− 0.07	(0.24)	0.00	(0.62)
Candy store										
1998 (/km^2^)										
0 (ref)										
> 0	− 0.01	(0.50)	0.02	(0.59)	0.06	(0.73)	− 0.04	(0.53)	− 1.89	(2.23)
1998–2007										
Decreased	− 0.36	(0.59)	− 0.34	(0.70)	− 0.32	(0.85)	− 0.51	(0.62)	2.55	(2.39)
Constant (ref)										
Increased	0.11	(0.33)	− 0.10	(0.39)	0.33	(0.47)	0.01	(0.35)	0.61	(0.90)
Fruit and vegetable market										
1998 (/km^2^)										
0 (ref)										
> 0	0.30	(0.49)	0.14	(0.56)	0.52	(0.73)	0.46	(0.52)	0.55	(1.60)
1998–2007										
Decreased	0.45	(0.61)	1.19	(0.74)	− 0.32	(0.89)	0.38	(0.64)	− 0.98	(2.20)
Constant (ref)										
Increased	− 0.09	(0.31)	− 0.02	(0.36)	− 0.32	(0.47)	0.00	(0.34)	− 0.92	(0.97)
Meat and fish market										
1998 (/km^2^)										
0 (ref)										
> 0	0.22	(0.32)	0.42	(0.37)	− 0.09	(0.47)	0.30	(0.36)	− 0.19	(0.81)
1998–2007										
Decreased	− 0.28	(0.42)	− 0.60	(0.49)	0.16	(0.63)	− 0.27	(0.45)	− 1.23	(1.51)
Constant (ref)										
Increased	0.20	(0.25)	− 0.16	(0.29)	0.63	(0.37)	0.16	(0.27)	0.32	(0.80)
Beverage store										
1998 (/km^2^)										
0 (ref)										
> 0	− 0.50	(0.38)	− 0.49	(0.45)	− 0.36	(0.54)	− 0.55	(0.41)	− 0.18	(1.20)
1998–2007										
Decreased	0.31	(0.50)	0.14	(0.58)	0.47	(0.72)	0.35	(0.52)	− 2.15	(3.15)
Constant (ref)										
Increased	− 0.21	(0.20)	− 0.15	(0.23)	− 0.32	(0.29)	− 0.25	(0.22)	− 0.38	(0.53)

All models were adjusted for age, gender, race/ethnicity, socioeconomic status, parental education, and urbanicity. Italicized numbers indicate statistical significance (*p < 0.05, **p < 0.01, ***p < 0.001)

The effects on child BMI of the changes in some other types of food outlet in school neighborhoods were found at different scales of analyses. For example, within 800-m road-network buffer zones, a higher BMI was found in urban schoolchildren who had experienced an increase of full-service restaurants in their school neighborhoods (*β* = 0.41, *p* < 0.05), in boys with an increase of convenience stores (*β* = 0.44, *p* < 0.05) and a decrease of fruit/vegetable markets (*β* = 1.85, *p* < 0.05), and in girls with the increase of fast-food restaurants (*β* = 0.70, *p* < 0.01) and meat/fish markets (*β* = 1.05, *p* < 0.05) (Additional file 1: Table S3). Consistently, the decrease of meat/fish markets was also associated with lower BMI in girls (*β* = − 0.74, *p* < 0.05) within school ZIP codes, where, in addition, the decrease of health/dietetic food stores was associated with the increased BMI (*β* = 0.49, *p* < 0.05), especially in girls (*β* = 0.94, *p* < 0.01) and non-urban schoolchildren (*β* = 1.50, *p* < 0.01) (Additional file 1: Table S6).

### Associations of school neighborhood food environments and child weight status

The longitudinal associations of the changes in school neighborhood food environments with child overweight/obesity risk were mainly found in non-urban schoolchildren and girls (Table 4). The higher overweight/obesity risk in non-urban schoolchildren was associated with the increase of convenience stores (OR = 1.46 [95% CI = 1.10–1.95]) and health/dietetic food stores (OR = 1.47 [95% CI = 1.00–2.15]). The lower overweight/obesity risk among girls was associated with an increase of dairy product stores (OR = 0.71 [95% CI = 0.54–0.92]) and a decrease of fruit/vegetable stores (OR = 0.42 [95% CI = 0.20–0.90]), while a higher overweight/obesity risk was associated with an increase of candy stores (OR = 1.50 [95% CI = 1.03–2.20]) and a decrease of beverage stores (OR = 2.61 [95% CI = 1.46–4.66]); similar associations of dairy product stores and the other three types of food outlet with the overweight/obesity risk in girls were also observed within school ZIP codes (Additional file 1: Table S7) and within 800-m road-network buffer zones around schools (Additional file 1: Table S4), respectively.

**Table 4:** Associations (odds ratio and 95% confidence interval) of changes in school neighborhood food environments within 800-m straight-line buffer zones during 1998–2007 with child overweight and obesity in 2007

Food outlet type	All (*n* = 7530)		Boys (*n* = 3780)		Girls (*n* = 3750)		Urban (*n* = 5040)		Non-urban (*n* = 2490)	
Supermarket										
1998 (/km^2^)										
0 (ref)										
> 0	1.13	[0.96, 1.34]	1.12	[0.88, 1.42]	1.18	[0.93, 1.50]	1.16	[0.94, 1.43]	1.16	[0.85, 1.58]
1998–2007										
Decreased	0.91	[0.74, 1.12]	0.95	[0.73, 1.24]	0.84	[0.62, 1.14]	0.99	[0.76, 1.28]	0.76	[0.50, 1.16]
Constant (ref)										
Increased	1.10	[0.94, 1.29]	1.09	[0.88, 1.33]	1.11	[0.89, 1.40]	1.10	[0.92, 1.33]	1.02	[0.74, 1.42]
Convenience store										
1998 (/km^2^)										
0 (ref)										
> 0	0.96	[0.82, 1.12]	0.92	[0.74, 1.13]	1.02	[0.81, 1.27]	0.92	[0.77, 1.10]	1.14	[0.80, 1.62]
1998–2007										
Decreased	1.13	[0.92, 1.39]	1.13	[0.86, 1.50]	1.14	[0.85, 1.52]	1.17	[0.90, 1.51]	1.00	[0.68, 1.48]
Constant (ref)										
Increased	1.05	[0.90, 1.22]	0.98	[0.80, 1.21]	1.15	[0.92, 1.43]	0.98	[0.81, 1.17]	1.46**	[1.10, 1.95]
Full-service restaurant										
1998 (/km^2^)										
0 (ref)										
> 0	0.89	[0.74, 1.06]	0.80	[0.63, 1.01]	0.98	[0.76, 1.26]	0.97	[0.77, 1.22]	0.75	[0.56, 1.02]
1998–2007										
Decreased	1.02	[0.83, 1.27]	1.08	[0.82, 1.43]	1.00	[0.74, 1.36]	0.99	[0.75, 1.31]	1.11	[0.80, 1.54]
Constant (ref)										
Increased	0.86	[0.73, 1.01]	0.81	[0.65, 1.01]	0.92	[0.73, 1.16]	0.88	[0.72, 1.09]	0.76	[0.58, 1.01]
Fast-food restaurant										
1998 (/km^2^)										
0 (ref)										
> 0	1.14	[0.97, 1.34]	1.20	[0.95, 1.51]	1.08	[0.85, 1.37]	1.15	[0.95, 1.38]	1.03	[0.73, 1.43]
1998–2007										
Decreased	1.03	[0.82, 1.30]	1.27	[0.94, 1.73]	0.82	[0.58, 1.15]	0.97	[0.75, 1.25]	1.43	[0.78, 2.63]
Constant (ref)										
Increased	1.02	[0.87, 1.18]	1.09	[0.88, 1.36]	0.93	[0.75, 1.17]	1.11	[0.93, 1.33]	0.84	[0.63, 1.13]
Retail bakery										
1998 (/km^2^)										
0 (ref)										
> 0	1.17	[0.96, 1.44]	1.23	[0.96, 1.59]	1.11	[0.83, 1.49]	1.16	[0.93, 1.45]	0.94	[0.53, 1.68]
1998–2007										
Decreased	0.86	[0.67, 1.10]	0.95	[0.69, 1.31]	0.78	[0.53, 1.15]	0.84	[0.64, 1.11]	1.37	[0.71, 2.64]
Constant (ref)										
Increased	0.85	[0.72, 1.02]	0.89	[0.69, 1.14]	0.81	[0.64, 1.03]	0.83	[0.68, 1.01]	1.02	[0.68, 1.53]
Dairy product store										
1998 (/km^2^)										
0 (ref)										
> 0	0.78	[0.60, 1.01]	0.67*	[0.48, 0.94]	0.90	[0.62, 1.31]	0.79	[0.61, 1.01]	0.65	[0.18, 2.27]
1998–2007										
Decreased	1.26	[0.87, 1.83]	1.50	[0.94, 2.39]	1.01	[0.58, 1.74]	1.27	[0.87, 1.86]	1.45	[0.32, 6.68]
Constant (ref)										
Increased	0.89	[0.74, 1.06]	1.08	[0.85, 1.38]	0.71**	[0.54, 0.92]	0.87	[0.71, 1.07]	0.90	[0.60, 1.35]
Health food store										
1998 (/km^2^)										
0 (ref)										
> 0	1.11	[0.89, 1.39]	1.10	[0.81, 1.51]	1.14	[0.81, 1.60]	1.05	[0.82, 1.36]	1.83**	[1.17, 2.86]
1998–2007										
Decreased	1.00	[0.75, 1.33]	0.98	[0.65, 1.46]	0.99	[0.62, 1.58]	1.01	[0.73, 1.40]	0.74	[0.33, 1.68]
Constant (ref)										
Increased	1.06	[0.88, 1.28]	0.94	[0.73, 1.21]	1.21	[0.92, 1.58]	1.00	[0.81, 1.25]	1.47*	[1.00, 2.15]
Candy store										
1998 (/km^2^)										
0 (ref)										
> 0	1.29	[0.85, 1.95]	1.09	[0.64, 1.87]	1.55	[0.82, 2.91]	1.19	[0.76, 1.85]	1.35	[0.34, 5.42]
1998–2007										
Decreased	0.87	[0.52, 1.45]	1.04	[0.54, 2.00]	0.73	[0.33, 1.61]	0.85	[0.48, 1.52]	1.25	[0.26, 5.95]
Constant (ref)										
Increased	1.18	[0.87, 1.61]	0.90	[0.59, 1.36]	1.50*	[1.03, 2.20]	1.10	[0.80, 1.52]	1.70	[0.82, 3.50]
Fruit and vegetable market										
1998 (/km^2^)										
0 (ref)										
> 0	1.34	[0.97, 1.85]	1.27	[0.81, 1.98]	1.52	[0.86, 2.68]	1.43*	[1.01, 2.03]	1.28	[0.55, 2.95]
1998–2007										
Decreased	0.71	[0.44, 1.16]	1.21	[0.64, 2.28]	0.42*	[0.20, 0.90]	0.68	[0.40, 1.15]	0.79	[0.27, 2.32]
Constant (ref)										
Increased	0.89	[0.68, 1.17]	1.05	[0.74, 1.47]	0.73	[0.47, 1.14]	0.96	[0.73, 1.28]	0.67	[0.26, 1.74]
Meat and fish market										
1998 (/km^2^)										
0 (ref)										
> 0	0.87	[0.67, 1.12]	1.01	[0.72, 1.42]	0.74	[0.48, 1.14]	0.89	[0.65, 1.21]	0.67	[0.39, 1.14]
1998–2007										
Decreased	0.96	[0.66, 1.39]	0.79	[0.50, 1.26]	1.14	[0.62, 2.08]	0.93	[0.62, 1.41]	1.15	[0.60, 2.21]
Constant (ref)										
Increased	0.99	[0.77, 1.28]	0.85	[0.62, 1.15]	1.17	[0.83, 1.65]	0.97	[0.75, 1.27]	0.85	[0.41, 1.76]
Beverage store										
1998 (/km^2^)										
0 (ref)										
> 0	0.74	[0.55, 1.01]	0.87	[0.58, 1.31]	0.66	[0.43, 1.02]	0.77	[0.55, 1.08]	0.52	[0.20, 1.31]
1998–2007										
Decreased	1.50	[0.97, 2.32]	0.87	[0.47, 1.61]	2.61**	[1.46, 4.66]	1.42	[0.90, 2.26]	0.73	[0.14, 3.85]
Constant (ref)										
Increased	1.07	[0.90, 1.27]	1.07	[0.87, 1.33]	1.03	[0.80, 1.33]	1.05	[0.86, 1.29]	1.09	[0.73, 1.63]

All models were adjusted for age, gender, race/ethnicity, socioeconomic status, parental education, and urbanicity. Italicized numbers indicate statistical significance (*p < 0.05, **p < 0.01, ***p < 0.001)

The higher obesity risk was associated with the increase of fast-food restaurants in urban schoolchildren (OR = 1.27 [95% CI = 1.02–1.59]) and of convenience stores in girls (OR = 1.41 [95% CI = 1.09–1.82]) and non-urban schoolchildren (OR = 1.60 [95% CI = 1.10–2.33]) (Table 5). The increase of full-service restaurants was related to lower obesity risk in boys (OR = 0.74 [95% CI = 0.57–0.95]). Consistently, the decrease of full-service restaurants was related to higher obesity risk in boys (OR = 1.45 [95% CI = 1.01–2.09]) within 800-m road-network buffer zones around schools (Additional file 1: Table S5). The schoolchildren who had experienced the decrease of dairy product stores showed the higher obesity risk (OR = 1.68 [95% CI = 1.07–2.65]), especially boys (OR = 2.92 [95% CI = 1.58–5.40]) and urban schoolchildren (OR = 1.67 [95% CI = 1.07–2.61]), which were also observed within 800-m road-network buffer zones around schools (Additional file 1: Table S5). The girls exposed to the increase of dairy product stores consistently showed the lower obesity risk (OR = 0.71 [95% CI = 0.51–0.98]). In addition, schoolchildren exposed to the decrease of meat/fish markets showed the lower obesity risk (OR = 0.57 [95% CI = 0.35–0.91]), especially urban schoolchildren (OR = 0.53 [95% CI = 0.32–0.87]). No similar results were found from the analyses within school ZIP codes (Additional file 1: Table S8).

**Table 5:** Associations (odds ratio and 95% confidence interval) of changes in school neighborhood food environments within 800-m straight-line buffer zones during 1998–2007 with child obesity in 2007

Food outlet type	All (*n* = 7530)		Boys (*n* = 3780)		Girls (*n* = 3750)		Urban (*n* = 5040)		Non-urban (*n* = 2490)	
Supermarket										
1998 (/km^2^)										
0 (ref)										
> 0	1.07	[0.88, 1.29]	1.10	[0.86, 1.41]	1.07	[0.81, 1.42]	1.09	[0.87, 1.36]	1.02	[0.71, 1.46]
1998–2007										
Decreased	0.88	[0.69, 1.12]	0.92	[0.67, 1.26]	0.76	[0.53, 1.09]	0.90	[0.67, 1.20]	0.96	[0.56, 1.65]
Constant (ref)										
Increased	0.99	[0.82, 1.19]	1.05	[0.82, 1.35]	0.90	[0.69, 1.17]	1.01	[0.82, 1.25]	0.98	[0.65, 1.47]
Convenience store										
1998 (/km^2^)										
0 (ref)										
> 0	1.01	[0.84, 1.22]	0.91	[0.71, 1.16]	1.11	[0.84, 1.47]	0.96	[0.77, 1.19]	1.05	[0.66, 1.69]
1998–2007										
Decreased	1.04	[0.79, 1.36]	1.22	[0.88, 1.68]	0.87	[0.56, 1.34]	1.12	[0.81, 1.56]	0.87	[0.51, 1.49]
Constant (ref)										
Increased	1.11	[0.92, 1.33]	0.97	[0.77, 1.23]	1.41**	[1.09, 1.82]	0.96	[0.77, 1.20]	1.60*	[1.10, 2.33]
Full-service restaurant										
1998 (/km^2^)										
0 (ref)										
> 0	0.94	[0.75, 1.17]	0.88	[0.65, 1.17]	0.99	[0.72, 1.37]	0.97	[0.74, 1.27]	0.93	[0.61, 1.44]
1998–2007										
Decreased	1.03	[0.79, 1.35]	0.95	[0.68, 1.34]	1.25	[0.85, 1.83]	1.02	[0.73, 1.42]	1.08	[0.68, 1.70]
Constant (ref)										
Increased	0.83	[0.67, 1.02]	0.74*	[0.57, 0.95]	0.98	[0.72, 1.33]	0.82	[0.64, 1.06]	0.79	[0.52, 1.21]
Fast-food restaurant										
1998 (/km^2^)										
0 (ref)										
> 0	0.96	[0.78, 1.18]	1.05	[0.81, 1.37]	0.87	[0.64, 1.20]	0.95	[0.76, 1.19]	1.07	[0.63, 1.84]
1998–2007										
Decreased	1.18	[0.86, 1.61]	1.23	[0.81, 1.86]	1.12	[0.72, 1.75]	1.18	[0.84, 1.66]	1.02	[0.44, 2.37]
Constant (ref)										
Increased	1.06	[0.88, 1.29]	1.05	[0.82, 1.35]	1.06	[0.80, 1.41]	1.27*	[1.02, 1.59]	0.72	[0.46, 1.12]
Retail bakery										
1998 (/km^2^)										
0 (ref)										
> 0	1.04	[0.81, 1.34]	1.02	[0.74, 1.42]	1.05	[0.72, 1.54]	0.90	[0.70, 1.16]	1.65	[0.67, 4.07]
1998–2007										
Decreased	0.91	[0.65, 1.26]	0.86	[0.56, 1.32]	0.94	[0.57, 1.56]	1.01	[0.72, 1.42]	1.07	[0.38, 3.02]
Constant (ref)										
Increased	0.93	[0.74, 1.16]	1.12	[0.84, 1.49]	0.74	[0.53, 1.03]	0.91	[0.72, 1.14]	1.13	[0.56, 2.28]
Dairy product store										
1998 (/km^2^)										
0 (ref)										
> 0	0.76	[0.54, 1.06]	0.50**	[0.32, 0.77]	1.22	[0.81, 1.85]	0.85	[0.62, 1.16]	0.23	[0.04, 1.49]
1998–2007										
Decreased	1.68*	[1.07, 2.65]	2.92***	[1.58, 5.40]	0.79	[0.43, 1.46]	1.67*	[1.07, 2.61]	2.49	[0.27, 22.76]
Constant (ref)										
Increased	0.91	[0.73, 1.13]	1.06	[0.80, 1.40]	0.71*	[0.51, 0.98]	0.89	[0.70, 1.14]	0.86	[0.51, 1.45]
Health food store										
1998 (/km^2^)										
0 (ref)										
> 0	0.92	[0.69, 1.23]	1.05	[0.73, 1.51]	0.77	[0.51, 1.17]	0.95	[0.70, 1.30]	0.86	[0.42, 1.74]
1998–2007										
Decreased	0.96	[0.65, 1.42]	0.88	[0.52, 1.49]	1.07	[0.63, 1.85]	0.92	[0.61, 1.40]	1.01	[0.41, 2.46]
Constant (ref)										
Increased	1.09	[0.87, 1.38]	1.02	[0.75, 1.38]	1.22	[0.89, 1.68]	1.16	[0.90, 1.50]	1.06	[0.64, 1.76]
Candy store										
1998 (/km^2^)										
0 (ref)										
> 0	0.89	[0.52, 1.52]	0.95	[0.46, 1.96]	0.83	[0.42, 1.66]	0.75	[0.43, 1.29]	1.00	[0.20, 4.97]
1998–2007										
Decreased	0.99	[0.53, 1.87]	1.22	[0.52, 2.89]	0.75	[0.33, 1.73]	1.12	[0.58, 2.16]	1.30	[0.19, 8.96]
Constant (ref)										
Increased	1.18	[0.86, 1.63]	1.43	[0.93, 2.20]	0.87	[0.54, 1.40]	1.13	[0.78, 1.64]	1.44	[0.80, 2.61]
Fruit and vegetable market										
1998 (/km^2^)										
0 (ref)										
> 0	1.23	[0.75, 2.01]	1.20	[0.64, 2.23]	1.22	[0.57, 2.62]	1.31	[0.76, 2.25]	1.18	[0.38, 3.68]
1998–2007										
Decreased	0.78	[0.43, 1.44]	0.85	[0.35, 2.08]	0.75	[0.31, 1.80]	0.73	[0.38, 1.40]	0.58	[0.13, 2.61]
Constant (ref)										
Increased	1.05	[0.76, 1.46]	1.05	[0.73, 1.51]	1.00	[0.60, 1.66]	1.24	[0.92, 1.66]	0.48	[0.13, 1.72]
Meat and fish market										
1998 (/km^2^)										
0 (ref)										
> 0	1.13	[0.83, 1.52]	1.06	[0.70, 1.60]	1.16	[0.71, 1.89]	1.16	[0.85, 1.58]	0.82	[0.28, 2.42]
1998–2007										
Decreased	0.57*	[0.35, 0.91]	0.60	[0.32, 1.11]	0.59	[0.31, 1.12]	0.53*	[0.32, 0.87]	0.78	[0.24, 2.57]
Constant (ref)										
Increased	0.98	[0.74, 1.31]	1.06	[0.76, 1.47]	0.86	[0.55, 1.35]	0.99	[0.74, 1.33]	1.04	[0.34, 3.21]
Beverage store										
1998 (/km^2^)										
0 (ref)										
> 0	0.89	[0.62, 1.28]	0.96	[0.61, 1.52]	0.89	[0.50, 1.60]	0.84	[0.56, 1.25]	1.14	[0.49, 2.63]
1998–2007										
Decreased	1.14	[0.65, 2.01]	1.18	[0.61, 2.28]	1.03	[0.46, 2.28]	1.21	[0.67, 2.18]	1.00	[1.00, 1.00]
Constant (ref)										
Increased	1.11	[0.89, 1.37]	1.09	[0.83, 1.44]	1.05	[0.76, 1.46]	1.10	[0.87, 1.39]	0.88	[0.51, 1.53]

All models were adjusted for age, gender, race/ethnicity, socioeconomic status, parental education, and urbanicity. Italicized numbers indicate statistical significance (*p < 0.05, **p < 0.01, ***p < 0.001)

## Discussion

This is a large-scale longitudinal study using nationally representative data from the USA to investigate the relationships between school neighborhood food environments and children’s weight status at three geographic scales. We found that (1) increased exposure to convenience stores and meat/vegetable markets in school vicinity was mainly associated with schoolchildren’s higher weight status, and increased exposure to fast-food restaurants, health/dietetic food stores, candy stores, and fruit/vegetable markets was associated with their higher weight status only in some gender- and urbanicity-specific subgroups; (2) increased exposure to dairy product stores in school vicinity was mainly associated with schoolchildren’s lower weight status, and increased exposure to full-service restaurants and beverage stores was associated with the lower weight status only in some gender- and urbanicity-specific subgroups; (3) findings from the analyses within 800-m straight-line buffer zones of schools were more consistent with our theory-based hypotheses than those from the analyses within 800-m road-network buffer zones of schools and school ZIP codes.

Given the previous limited and mixed findings at different local scales [20, 21, 29], it is imperative to conduct a large-scale study to deepen our understanding of the roles of different food venues around schools in the obesity epidemic. Most of the previous studies have focused on common food venues (e.g., supermarkets, grocery store, fast-food restaurants) [20–22, 29]. It has been suggested that simultaneously accounting for multiple types of healthy and unhealthy food outlets could yield more precise estimates of health effects than when considering only a small number of dimensions of the food environment [32–35]. Some types of food outlet are sparsely distributed in the USA and even more sparsely in school vicinity, such as retail bakery, fruit/vegetable market, candy store, and dairy product store. Hence, the associations between those food outlets and child obesity have been little examined in local studies due to insufficient study samples and/or variability in exposure to such food environments. Given the increasing trend of all those types of food venue over the 9-year period across the country, understanding their association with population weight status, although possibly confounded to some extent, is important for urban and land-use planning in the future.

In addition to adding new knowledge to this field, as many food items are provided in more than one type of food outlet, including those sparsely distributed food outlets (i.e., controlling for these variables) may in turn produce more reliable evidence on the associations between common food outlets and obesity risk. Most types of food venue provide a variety of foods, both healthy and unhealthy. Candy, for example, provided in supermarkets (normally considered as a healthy venue), would be classified as unhealthy when housed in a separate venue. With candy stores and other types of food outlet providing supermarket food categories included in our models, the commonly accepted association between greater access to supermarkets and higher weight status was not found in this study. Likewise, the venues classified as convenience stores may also provide healthy options, and the food variety in convenience stores is more varying across regions than in supermarkets (usually chain stores). Therefore, more local studies with the involvement of field validation are needed to investigate the relationships between some types of food venue and child obesity. In addition, the actual food acquisition and consumption should be somehow considered to elucidate unknown pathways from food environments to child weight status. The association between less access to dairy product stores and schoolchildren’s higher weight status found in this study, for example, may be partly attributed to compensatory eating behaviors, which in particular matter in the regions where food is scarce [19].

Some gender-specific associations might be attributed to different social or eating behaviors, which, however, need more ancillary data to substantiate these links. For example, only girls with increased exposure to candy stores showed higher overweight/obesity risk. Additionally, fruit/vegetable markets are usually available in a more mobile form, which may take place only during certain times of a day on certain days of a week (e.g., a farmer’s market). Previous studies have reported failure of on-site validation for this category [36]. Due to our national study design, we were only able to conduct a visual validation in Google Maps for a limited sample of records, during which we either failed to find fruit/vegetable stands. Also, availability is not equal to consumption. These reasons may underlie some of the counterintuitive findings of this study, including the positive association between the exposure to fruit/vegetable markets and health/dietetic food stores and children’s overweight/obesity risk in non-urban regions. Thus, these results should be interpreted with caution.

An important advantage of undertaking this study at multiple geographic scales is to inform future studies that finding of food environmental research at one scale may not be sufficient, due partly to the uncertain location of schools (or individuals) within ZIP codes. Results from the analyses within school road-network buffer zones were not best aligned with our theory-based hypotheses, which further implies that GIS-based objective measures of food environments may not substitute for other types of measures, such as the perceptions of the neighborhood food environment, which could represent how individuals perceive their surroundings and consequently make decisions on how they actually interact with the surroundings. For example, the ecological momentary assessment (EMA) [37], where individuals are prompted to answer short surveys on their smartphone about the decision-making process related to a health behavior at a specific moment in time, is a commonly used methodology in spatial lifecourse epidemiology to complement objective measures of built and food environments [38].

This study has some limitations that highlight desirable future research directions. First, the classification of food venues needs to be refined. Due to the limited number of children relative to a wide range of food outlets of interest, we did not differentiate many detailed categories of food outlets represented by six-digit or eight-digit SIC codes. This prevented us from discriminating the effects of distinct types of food outlet falling under one main category, such as seafood and pizza restaurants in full-service restaurants and tea and soft drinks in beverage stores (Additional file 1: Table S1). However, simply using six-digit or eight-digit SIC codes cannot easily solve this problem, as a six-digit category still includes both healthy and unhealthy venues, and a venue in an eight-digit category still provides both healthy and unhealthy food. More work is needed to untangle these complexities, e.g., the inclusion of household surveys and individual purchasing and consumption data [19].

Second, the accuracy of the D&B data needs more ground-truthing work or virtual/remote assessment tools to validate it. In addition to changes in geographic locations, some entities might have experienced changes in primary markets or become closed during our 9-year study period. Therefore, some non-spatial information, such as business start-ups and failures, should be recorded in D&B datasets in better quality than currently, and hence be used to construct more robust indicators of food environments. In addition, despite no significant differences in sociodemographic characteristics and weight status between the included and excluded schoolchildren and no evidence showing that lost to follow-up is not random, the findings of this study should be generalized to the entire US young population with caution. Different multiple imputation methods will be used to deal with missing data and compare with the findings of this study in future efforts.

Third, in addition to using pre-defined administrative or geographic units, the “school neighborhood” boundary needs to be delineated with consideration of the routes and ways of commuting between home and school. By doing so, individual exposure to the food environment around schools could be measured more accurately [39–41]. The food environment and relevant regulations within schools should also be considered, which could possibly help to explain students’ purchasing, consumption, and compensatory eating behaviors [19]. We are also aware that children’s realistic interactions with food environments in the vicinity of their schools may be affected by age, home-to-school distance, availability of school lunch programs and on-campus vending machines, and school bus and after-school programs. Therefore, this study was limited by the unavailability of individuals’ residential addresses and school food-related policies.

Finally, we only focused on the effect of school neighborhood food environments without intention to be inclusive with all potential predictors added in. The results should be interpreted with caution as the significant relationship is not free of potential estimation issues due to possible omitted variables and measurement errors in the models, which, however, is impossible to completely avoid in regression analysis.

## Conclusion

This study revealed the relationships between school neighborhood food environments and children’s BMI and obesity risk over a 9-year follow-up period in a US nationally representative study. It suggests the potential benefit of improving food environments around schools for preventing childhood obesity. Three national spatial datasets (food outlet, road network, and ZIP code boundary) were processed to match with national survey data, which is an exemplary study in spatial lifecourse epidemiology [38]. It has important public health implications in terms of both school-based dietary intervention design and urban planning for the future (e.g., considering effects of the spatial configuration of various food outlets in school neighborhoods on children’s dietary behaviors and weight status and interventions to be developed). Survey and consumer purchasing data, as well as school environments and policies, could be integrated into future research to unravel the mechanisms of how school neighborhood food environments affect individual and family purchasing behaviors.

## Additional file

Additional file 1: Supplementary appendices including Standard Industrial Classification (SIC) codes used in the Dun and Bradstreet (D&B) datasets, a comparison of the characteristics of included and excluded schoolchildren, and sensitivity analyses. (DOCX 102 kb)

## Abbreviations

BMI: Body mass index
D&B: Dun and Bradstreet
ECLS-K: Early Childhood Longitudinal Study–Kindergarten
EMA: Ecological momentary assessment
SES: Socioeconomic status
SIC: Standard Industrial Classification
