# Supplementary material for: Effects of school neighborhood food environments on childhood obesity at multiple scales: a longitudinal kindergarten cohort study in the USA
Source: BMC Med. 2019 May 22;17:99. doi: 10.1186/s12916-019-1329-2 (PMC6532159; doi:10.1186/s12916-019-1329-2)
Supplement: Supplementary file 1 — Supplementary appendices including Standard Industrial Classification (SIC) codes used in the Dun and Bradstreet (D&B) datasets, a comparison of the characteristics of included and excluded schoolchildren, and sensitivity analyses. (DOCX 102 kb) [file 12916_2019_1329_MOESM1_ESM.docx]

**Table S1.** Hierarchical Standard Industrial Classification (SIC) codes used in the Dun and Bradstreet (D&B) commercial datasets.

| 4-digit | | 6-digit^*^ | | 8-digit^**^ | |
| --- | --- | --- | --- | --- | --- |
| 5411 | Grocery stores | 541101 | Supermarkets | 54110101 | Supermarkets, chain |
|  |  |  |  | 54110102 | Supermarkets, greater than 100,000 ft^2^ (hypermarket) |
|  |  |  |  | 54110103 | Supermarkets, independent |
|  |  |  |  | 54110104 | Supermarkets, 55,000 - 65,000 ft^2^ (superstore) |
|  |  |  |  | 54110105 | Supermarkets, 66,000 - 99,000 ft^2^ |
|  |  | 541102 | Convenience stores | 54110201 | Convenience stores, chain |
|  |  |  |  | 54110202 | Convenience stores, independent |
|  |  | 541199 | Grocery stores, nec | 54119901 | Cooperative food stores |
|  |  |  |  | 54119902 | Delicatessen stores |
|  |  |  |  | 54119903 | Frozen food and freezer plans, except meat |
|  |  |  |  | 54119904 | Grocery stores, chain |
|  |  |  |  | 54119905 | Grocery stores, independent |
| 5421 | Meat and fish markets | 542101 | Fish and seafood markets | 54210101 | Fish markets |
|  |  |  |  | 54210102 | Seafood markets |
|  |  | 542102 | Meat markets, including freezer provisioners | 54210201 | Food and freezer plans, meat |
|  |  |  |  | 54210202 | Freezer provisioners, meat |
| 5431 | Fruit and vegetable markets | 543199 | Fruit and vegetable markets, nec | 54319901 | Fruit stands or markets |
|  |  |  |  | 54319902 | Vegetable stands or markets |
| 5441 | Candy, nut, and confectionery stores | 544199 | Candy, nut, and confectionery stores, nec | 54419901 | Candy |
|  |  |  |  | 54419902 | Confectionery |
|  |  |  |  | 54419903 | Confectionery produced for direct sale on the premises |
|  |  |  |  | 54419904 | Nuts |
|  |  |  |  | 54419905 | Popcorn, including caramel corn |
| 5451 | Dairy products stores | 545199 | Dairy products stores, nec | 54519901 | Butter |
|  |  |  |  | 54519902 | Cheese |
|  |  |  |  | 54519903 | Ice cream (packaged) |
|  |  |  |  | 54519904 | Milk |
| 5461 | Retail bakeries | 546199 | Retail bakeries, nec | 54619901 | Bagels |
|  |  |  |  | 54619902 | Bread |
|  |  |  |  | 54619903 | Cakes |
|  |  |  |  | 54619904 | Cookies |
|  |  |  |  | 54619905 | Doughnuts |
|  |  |  |  | 54619906 | Pastries |
|  |  |  |  | 54619907 | Pies |
|  |  |  |  | 54619908 | Pretzels |
| 5499 | Miscellaneous food stores | 549901 | Health and dietetic food stores | 54990101 | Dietetic foods |
|  |  |  |  | 54990102 | Health foods |
|  |  |  |  | 54990103 | Vitamin food stores |
|  |  | 549902 | Beverage stores | 54990201 | Coffee |
|  |  |  |  | 54990202 | Juices, fruit or vegetable |
|  |  |  |  | 54990203 | Soft drinks |
|  |  |  |  | 54990204 | Tea |
|  |  |  |  | 54990205 | Water: distilled mineral or spring |
|  |  | 549999 | Miscellaneous food stores, nec | 54999901 | Dried fruit |
|  |  |  |  | 54999902 | Eggs and poultry |
|  |  |  |  | 54999903 | Food gift baskets |
|  |  |  |  | 54999904 | Gourmet food stores |
|  |  |  |  | 54999905 | Spices and herbs |
| 5812 | Eating places (termed “full-service restaurants” in this study) | 581201 | Ethnic food restaurants | 58120101 | American restaurant |
|  |  |  |  | 58120102 | Cajun restaurant |
|  |  |  |  | 58120103 | Chinese restaurant |
|  |  |  |  | 58120104 | French restaurant |
|  |  |  |  | 58120105 | German restaurant |
|  |  |  |  | 58120106 | Greek restaurant |
|  |  |  |  | 58120107 | Indian/Pakistan restaurant |
|  |  |  |  | 58120108 | Italian restaurant |
|  |  |  |  | 58120109 | Japanese restaurant |
|  |  |  |  | 58120110 | Korean restaurant |
|  |  |  |  | 58120111 | Lebanese restaurant |
|  |  |  |  | 58120112 | Mexican restaurant |
|  |  |  |  | 58120113 | Spanish restaurant |
|  |  |  |  | 58120114 | Sushi bar |
|  |  |  |  | 58120115 | Thai restaurant |
|  |  |  |  | 58120116 | Vietnamese restaurant |
|  |  |  |  | 58120117 | Pakistani restaurant |
|  |  | 581202 | Ice cream, soft drink and soda fountain stands | 58120201 | Concessionaire |
|  |  |  |  | 58120202 | Frozen yogurt stand |
|  |  |  |  | 58120203 | Ice cream stands or dairy bars |
|  |  |  |  | 58120204 | Snow cone stand |
|  |  |  |  | 58120205 | Soda fountain |
|  |  |  |  | 58120206 | Soft drink stand |
|  |  | 581203 | Fast food restaurants and stands | 58120301 | Box lunch stand |
|  |  |  |  | 58120302 | Carry-out only (except pizza) restaurant |
|  |  |  |  | 58120303 | Chili stand |
|  |  |  |  | 58120304 | Coffee shop |
|  |  |  |  | 58120305 | Delicatessen (eating places) |
|  |  |  |  | 58120306 | Drive-in restaurant |
|  |  |  |  | 58120307 | Fast-food restaurant, chain |
|  |  |  |  | 58120308 | Fast-food restaurant, independent |
|  |  |  |  | 58120309 | Food bars |
|  |  |  |  | 58120310 | Grills (eating places) |
|  |  |  |  | 58120311 | Hamburger stand |
|  |  |  |  | 58120312 | Hot dog stand |
|  |  |  |  | 58120313 | Sandwiches and submarines shop |
|  |  |  |  | 58120314 | Snack bar |
|  |  |  |  | 58120315 | Snack shop |
|  |  | 581204 | Lunchrooms and cafeterias | 58120401 | Automat (eating places) |
|  |  |  |  | 58120402 | Cafeteria |
|  |  |  |  | 58120403 | Luncheonette |
|  |  |  |  | 58120404 | Lunchroom |
|  |  |  |  | 58120405 | Restaurant, lunch counter |
|  |  | 581205 | Family restaurants | 58120501 | Restaurant, family: chain |
|  |  |  |  | 58120502 | Restaurant, family: independent |
|  |  | 581206 | Pizza restaurants | 58120601 | Pizzeria, chain |
|  |  |  |  | 58120602 | Pizzeria, independent |
|  |  | 581207 | Seafood restaurants | 58120701 | Oyster bar |
|  |  |  |  | 58120702 | Seafood shack |
|  |  | 581208 | Steak and barbecue restaurants | 58120801 | Barbecue restaurant |
|  |  |  |  | 58120802 | Steak restaurant |
|  |  | 581299 | Eating places, nec | 58129901 | Buffet (eating places) |
|  |  |  |  | 58129902 | Cafe |
|  |  |  |  | 58129903 | Caterers |
|  |  |  |  | 58129904 | Chicken restaurant |
|  |  |  |  | 58129905 | Commissary restaurant |
|  |  |  |  | 58129906 | Contract food services |
|  |  |  |  | 58129907 | Diner |
|  |  |  |  | 58129908 | Dinner theater |
|  |  |  |  | 58129909 | Health food restaurant |

^*^nec = not elsewhere classified ^**^ft^2^ = square feet

Note: Each color represents a major category of food (underlined). Highlighted were excluded from this study. If a 6-digit category completely belongs to a 4-digit category, then all 8-digit categories under that 6-digit category are in black (no highlighting colors). If all 8-digit categories under a 6-digit category do not belong to one major category, that 6-digit category is in black.

**Table S2**. Socio-demographic characteristics at baseline and weight status at baseline and 5^th^ wave of ECLS-K in the included and excluded schoolchildren^a^

| **Variables** | **% or Mean ± SD** | | |  | *p*-value^b^ |
| --- | --- | --- | --- | --- | --- |
|  | **All**  (n=9,440) | **Included**  (n=7,530) | **Excluded**  (n=1,910) |  |  |
| **Socio-demographic characteristics** | | |  |  |  |
| *Age (years)* | 6.2±0.4 | 6.2±0.4 | 6.3±0.3 |  | 0.398 |
| *Gender* |  |  |  |  | 0.215 |
| Boy | 51.9 | 51.2 | 54.4 |  |  |
| Girl | 48.1 | 48.8 | 45.6 |  |  |
| *Race/ethnicity* |  |  |  |  | **<0.001** |
| White | 57.4 | 58.0 | 55.2 |  |  |
| Black | 17.1 | 17.5 | 15.3 |  |  |
| Hispanic | 18.1 | 18.2 | 17.7 |  |  |
| Asian | 3.0 | 2.9 | 3.4 |  |  |
| Others | 4.4 | 3.4 | 8.4 |  |  |
| *Parental education* |  |  |  |  | 0.105 |
| ≤ High school | 35.2 | 33.8 | 39.9 |  |  |
| Vocational/college | 31.9 | 32.5 | 29.6 |  |  |
| Bachelor | 18.8 | 19.2 | 17.3 |  |  |
| ≥ Graduate | 14.2 | 14.5 | 13.2 |  |  |
| *Household annual income ($)* |  |  |  |  | 0.703 |
| ≤30,000 | 35.5 | 35.0 | 38.1 |  |  |
| >30,000 but ≤50,000 | 23.8 | 24.1 | 22.5 |  |  |
| >50,000 but ≤75,000 | 18.3 | 18.4 | 18.0 |  |  |
| >75,000 | 22.3 | 22.5 | 21.5 |  |  |
| *Urbanicity* |  |  |  |  |  |
| Urban | 68.0 | 68.4 | 66.8 |  | 0.522 |
| Non-urban | 32.0 | 31.6 | 33.2 |  |  |
| **Weight status**^c^ |  |  |  |  |  |
| *BMI (kg/m^2^)* | 16.4±2.3 | 16.4±2.2 | 16.4±2.0 |  | 0.721 |
| *Overweight and obesity* | 26.2 | 26.6 | 24.8 |  | 0.419 |
| *Obesity* | 11.5 | 11.3 | 12.6 |  | 0.426 |

^a^ Sampling weights were used in the analyses.

^b^ *p*-values tested the differences in each variable between included and excluded samples and were based on χ^2^ tests for categorical variables or *t*-tests for continuous variables.

^c^ Children were classified as overweight and obesity if their gender-age-specific BMI ≥ 85^th^ and 95^th^ percentiles of the 2000 CDC Growth Chart, respectively.

**Table S3**. Associations (coefficient and standard error) of changes in school neighborhood food environments within 800-m road-network buffer zones during 1998-2007 with child body mass index in 2007^a^

| **Food outlet type** | **All**  (n=7,530) | | **Boy**  (n=3,780) | | **Girl**  (n=3,750) | | **Urban**  (n = 5,040) | | **Non-urban**  (n = 2,490) | |
| --- | --- | --- | --- | --- | --- | --- | --- | --- | --- | --- |
| **Supermarket** | | | |  |  |  |  |  |  |  |
| *1998* (/km^2^) |  |  |  |  |  |  |  |  |  |  |
| 0 (ref) |  |  |  |  |  |  |  |  |  |  |
| >0 | 0.06 | (0.21) | 0.07 | (0.24) | -0.02 | (0.30) | 0.03 | (0.25) | 0.11 | (0.44) |
| *1998-2007* |  |  |  |  |  |  |  |  |  |  |
| Decreased | -0.26 | (0.25) | -0.19 | (0.29) | -0.30 | (0.37) | -0.24 | (0.30) | -0.33 | (0.55) |
| Constant (ref) |  |  |  |  |  |  |  |  |  |  |
| Increased | 0.15 | (0.20) | 0.18 | (0.23) | 0.02 | (0.28) | 0.20 | (0.22) | 0.07 | (0.40) |
| **Convenience store** | | | |  |  |  |  |  |  |  |
| *1998* (/km^2^) |  |  |  |  |  |  |  |  |  |  |
| 0 (ref) |  |  |  |  |  |  |  |  |  |  |
| >0 | -0.11 | (0.22) | -0.33 | (0.26) | 0.17 | (0.32) | -0.26 | (0.25) | 0.36 | (0.54) |
| *1998-2007* |  |  |  |  |  |  |  |  |  |  |
| Decreased | 0.31 | (0.30) | 0.54 | (0.34) | 0.02 | (0.44) | 0.58 | (0.35) | -0.60 | (0.57) |
| Constant (ref) |  |  |  |  |  |  |  |  |  |  |
| Increased | 0.37 | (0.19) | **0.44^*^** | (0.22) | 0.38 | (0.28) | 0.41 | (0.23) | 0.19 | (0.37) |
| **Full-service restaurant** | | |  |  |  |  |  |  |  |  |
| *1998* (/km^2^) |  |  |  |  |  |  |  |  |  |  |
| 0 (ref) |  |  |  |  |  |  |  |  |  |  |
| >0 | -0.36 | (0.20) | -0.32 | (0.23) | -0.41 | (0.28) | -0.30 | (0.23) | -0.18 | (0.47) |
| *1998-2007* |  |  |  |  |  |  |  |  |  |  |
| Decreased | 0.38 | (0.25) | 0.33 | (0.29) | 0.42 | (0.36) | 0.47 | (0.29) | 0.01 | (0.52) |
| Constant (ref) |  |  |  |  |  |  |  |  |  |  |
| Increased | 0.22 | (0.17) | -0.01 | (0.20) | 0.43 | (0.25) | **0.41^*^** | (0.21) | -0.17 | (0.34) |
| **Fast-food restaurant** | | |  |  |  |  |  |  |  |  |
| *1998* (/km^2^) |  |  |  |  |  |  |  |  |  |  |
| 0 (ref) |  |  |  |  |  |  |  |  |  |  |
| >0 | -0.22 | (0.22) | -0.09 | (0.25) | -0.42 | (0.32) | -0.24 | (0.26) | -0.47 | (0.50) |
| *1998-2007* |  |  |  |  |  |  |  |  |  |  |
| Decreased | 0.09 | (0.32) | -0.13 | (0.37) | 0.34 | (0.46) | -0.01 | (0.35) | 0.90 | (0.81) |
| Constant (ref) |  |  |  |  |  |  |  |  |  |  |
| Increased | 0.33 | (0.18) | 0.03 | (0.21) | **0.70^**^** | (0.26) | 0.29 | (0.21) | 0.48 | (0.40) |
| **Retail bakery** | | |  |  |  |  |  |  |  |  |
| *1998* (/km^2^) |  |  |  |  |  |  |  |  |  |  |
| 0 (ref) |  |  |  |  |  |  |  |  |  |  |
| >0 | -0.09 | (0.26) | 0.06 | (0.30) | -0.07 | (0.38) | -0.17 | (0.29) | -0.24 | (0.72) |
| *1998-2007* |  |  |  |  |  |  |  |  |  |  |
| Decreased | -0.29 | (0.34) | -0.20 | (0.40) | -0.40 | (0.50) | -0.40 | (0.37) | 0.52 | (0.87) |
| Constant (ref) |  |  |  |  |  |  |  |  |  |  |
| Increased | -0.26 | (0.24) | -0.18 | (0.29) | -0.47 | (0.34) | -0.24 | (0.26) | 0.16 | (0.67) |
| **Dairy product store** | | | |  |  |  |  |  |  |  |
| *1998* (/km^2^) |  |  |  |  |  |  |  |  |  |  |
| 0 (ref) |  |  |  |  |  |  |  |  |  |  |
| >0 | -0.13 | (0.33) | -0.30 | (0.39) | -0.01 | (0.47) | -0.09 | (0.36) | -0.45 | (1.08) |
| *1998-2007* |  |  |  |  |  |  |  |  |  |  |
| Decreased | 0.87 | (0.47) | **1.76^**^** | (0.55) | -0.12 | (0.70) | **1.19^*^** | (0.50) | -1.38 | (1.53) |
| Constant (ref) |  |  |  |  |  |  |  |  |  |  |
| Increased | 0.12 | (0.26) | **0.60^*^** | (0.30) | -0.45 | (0.38) | 0.16 | (0.28) | -0.15 | (0.61) |
| **Health food store** | |  |  |  |  |  |  |  |  |  |
| *1998* (/km^2^) |  |  |  |  |  |  |  |  |  |  |
| 0 (ref) |  |  |  |  |  |  |  |  |  |  |
| >0 | 0.26 | (0.36) | 0.37 | (0.41) | 0.01 | (0.53) | 0.08 | (0.40) | 0.61 | (0.90) |
| *1998-2007* |  |  |  |  |  |  |  |  |  |  |
| Decreased | -0.57 | (0.46) | -0.84 | (0.55) | -0.11 | (0.66) | -0.50 | (0.50) | -0.23 | (1.25) |
| Constant (ref) |  |  |  |  |  |  |  |  |  |  |
| Increased | -0.36 | (0.26) | -0.29 | (0.31) | -0.33 | (0.38) | -0.38 | (0.29) | -0.44 | (0.62) |
| **Candy store** | | |  |  |  |  |  |  |  |  |
| *1998* (/km^2^) |  |  |  |  |  |  |  |  |  |  |
| 0 (ref) |  |  |  |  |  |  |  |  |  |  |
| >0 | 0.26 | (0.80) | 0.05 | (0.91) | 0.34 | (1.27) | 0.33 | (0.81) | 1.01 | (1.47) |
| *1998-2007* |  |  |  |  |  |  |  |  |  |  |
| Decreased | -0.59 | (0.87) | -0.10 | (0.99) | -0.96 | (1.37) | -0.71 | (0.89) | 0.00 | (.) |
| Constant (ref) |  |  |  |  |  |  |  |  |  |  |
| Increased | 0.27 | (0.37) | -0.04 | (0.44) | 0.66 | (0.52) | 0.26 | (0.41) | 0.47 | (0.86) |
| **Fruit and vegetable market** | | | |  |  |  |  |  |  |  |
| *1998* (/km^2^) |  |  |  |  |  |  |  |  |  |  |
| 0 (ref) |  |  |  |  |  |  |  |  |  |  |
| >0 | -0.15 | (0.56) | -0.36 | (0.65) | 0.22 | (0.83) | 0.15 | (0.58) | -5.14 | (2.76) |
| *1998-2007* |  |  |  |  |  |  |  |  |  |  |
| Decreased | 1.29 | (0.72) | **1.85^*^** | (0.88) | 0.48 | (1.05) | 1.14 | (0.75) | – | – |
| Constant (ref) |  |  |  |  |  |  |  |  |  |  |
| Increased | -0.03 | (0.37) | -0.21 | (0.43) | -0.01 | (0.56) | 0.10 | (0.40) | -2.29 | (1.24) |
| **Meat and fish market** | | | |  |  |  |  |  |  |  |
| *1998* (/km^2^) |  |  |  |  |  |  |  |  |  |  |
| 0 (ref) |  |  |  |  |  |  |  |  |  |  |
| >0 | -0.36 | (0.41) | 0.33 | (0.48) | -1.06 | (0.59) | -0.21 | (0.45) | -0.40 | (1.00) |
| *1998-2007* |  |  |  |  |  |  |  |  |  |  |
| Decreased | 0.46 | (0.51) | -0.51 | (0.60) | 1.43 | (0.76) | 0.35 | (0.55) | – | – |
| Constant (ref) |  |  |  |  |  |  |  |  |  |  |
| Increased | 0.44 | (0.29) | -0.14 | (0.35) | **1.05^*^** | (0.41) | 0.49 | (0.31) | -0.30 | (0.83) |
| **Beverage store** | | |  |  |  |  |  |  |  |  |
| *1998* (/km^2^) |  |  |  |  |  |  |  |  |  |  |
| 0 (ref) |  |  |  |  |  |  |  |  |  |  |
| >0 | -0.33 | (0.48) | -0.07 | (0.57) | -0.45 | (0.69) | -0.44 | (0.53) | 0.32 | (1.21) |
| *1998-2007* |  |  |  |  |  |  |  |  |  |  |
| Decreased | 0.26 | (0.60) | -0.16 | (0.71) | 0.75 | (0.88) | 0.38 | (0.64) | – | – |
| Constant (ref) |  |  |  |  |  |  |  |  |  |  |
| Increased | -0.41 | (0.23) | -0.18 | (0.28) | -0.55 | (0.33) | -0.49 | (0.27) | -0.48 | (0.51) |

^a^All models were adjusted for age, gender, race/ethnicity, socioeconomic status, parental education, and urbanicity. Boldfaced numbers indicate statistical significance (^*^*p*<0.05, ^**^*p*<0.01, ^***^*p*<0.001).

**Table S4**. Associations (odds ratio and 95% confidence interval) of changes in school neighborhood food environments within 800-m road-network buffer zones during 1998-2007 with child overweight and obesity in 2007^a^

| **Food outlet type** | **All**  (n=7,530) | | **Boy**  (n=3,780) | | **Girl**  (n=3,750) | | **Urban**  (n = 5,040) | | **Non-urban**  (n = 2,490) | |
| --- | --- | --- | --- | --- | --- | --- | --- | --- | --- | --- |
| **Supermarket** | | | |  |  |  |  |  |  |  |
| *1998* (/km^2^) |  |  |  |  |  |  |  |  |  |  |
| 0 (ref) |  |  |  |  |  |  |  |  |  |  |
| >0 | 1.20 | [0.99,1.45] | 1.14 | [0.87,1.49] | 1.26 | [0.96,1.64] | 1.22 | [0.98,1.52] | 1.25 | [0.89,1.75] |
| *1998-2007* |  |  |  |  |  |  |  |  |  |  |
| Decreased | 0.84 | [0.67,1.05] | 0.85 | [0.63,1.15] | 0.83 | [0.60,1.15] | 0.85 | [0.66,1.10] | 0.68 | [0.45,1.02] |
| Constant (ref) |  |  |  |  |  |  |  |  |  |  |
| Increased | 1.03 | [0.86,1.22] | 0.97 | [0.76,1.22] | 1.05 | [0.82,1.35] | 0.96 | [0.78,1.17] | 1.33 | [0.99,1.77] |
| **Convenience store** | | | |  |  |  |  |  |  |  |
| *1998* (/km^2^) |  |  |  |  |  |  |  |  |  |  |
| 0 (ref) |  |  |  |  |  |  |  |  |  |  |
| >0 | 1.04 | [0.86,1.25] | 0.89 | [0.70,1.12] | 1.24 | [0.93,1.65] | 0.98 | [0.80,1.21] | 1.34 | [0.88,2.06] |
| *1998-2007* |  |  |  |  |  |  |  |  |  |  |
| Decreased | 0.93 | [0.72,1.21] | 1.07 | [0.78,1.46] | 0.81 | [0.55,1.18] | 1.07 | [0.80,1.45] | 0.64 | [0.38,1.07] |
| Constant (ref) |  |  |  |  |  |  |  |  |  |  |
| Increased | 0.96 | [0.81,1.13] | 0.98 | [0.79,1.21] | 0.97 | [0.76,1.24] | 0.91 | [0.75,1.12] | 1.20 | [0.91,1.59] |
| **Full-service restaurant** | | |  |  |  |  |  |  |  |  |
| *1998* (/km^2^) |  |  |  |  |  |  |  |  |  |  |
| 0 (ref) |  |  |  |  |  |  |  |  |  |  |
| >0 | 0.88 | [0.74,1.04] | **0.77^*^** | [0.61,0.98] | 0.97 | [0.76,1.24] | 0.94 | [0.77,1.15] | **0.70^*^** | [0.50,0.98] |
| *1998-2007* |  |  |  |  |  |  |  |  |  |  |
| Decreased | 1.14 | [0.92,1.42] | 1.25 | [0.95,1.65] | 1.05 | [0.76,1.45] | 1.16 | [0.89,1.50] | 1.28 | [0.86,1.92] |
| Constant (ref) |  |  |  |  |  |  |  |  |  |  |
| Increased | 1.05 | [0.90,1.23] | 0.99 | [0.81,1.22] | 1.12 | [0.90,1.41] | 1.17 | [0.98,1.41] | 0.90 | [0.66,1.24] |
| **Fast-food restaurant** | | |  |  |  |  |  |  |  |  |
| *1998* (/km^2^) |  |  |  |  |  |  |  |  |  |  |
| 0 (ref) |  |  |  |  |  |  |  |  |  |  |
| >0 | 0.99 | [0.81,1.19] | 1.01 | [0.79,1.30] | 0.96 | [0.72,1.28] | 0.98 | [0.78,1.22] | 0.99 | [0.69,1.44] |
| *1998-2007* |  |  |  |  |  |  |  |  |  |  |
| Decreased | 1.10 | [0.84,1.46] | 1.37 | [0.94,1.99] | 0.86 | [0.57,1.29] | 1.12 | [0.82,1.53] | 0.93 | [0.50,1.73] |
| Constant (ref) |  |  |  |  |  |  |  |  |  |  |
| Increased | 1.11 | [0.95,1.30] | 1.13 | [0.91,1.41] | 1.11 | [0.88,1.40] | 1.16 | [0.97,1.40] | 0.87 | [0.64,1.18] |
| **Retail bakery** | | |  |  |  |  |  |  |  |  |
| *1998* (/km^2^) |  |  |  |  |  |  |  |  |  |  |
| 0 (ref) |  |  |  |  |  |  |  |  |  |  |
| >0 | 1.11 | [0.90,1.38] | 1.27 | [0.99,1.64] | 1.00 | [0.71,1.41] | 1.16 | [0.91,1.48] | **0.56^*^** | [0.33,0.95] |
| *1998-2007* |  |  |  |  |  |  |  |  |  |  |
| Decreased | 0.93 | [0.71,1.23] | 0.92 | [0.64,1.31] | 0.97 | [0.62,1.50] | 0.85 | [0.61,1.18] | **2.27^*^** | [1.15,4.51] |
| Constant (ref) |  |  |  |  |  |  |  |  |  |  |
| Increased | 0.85 | [0.68,1.06] | 0.86 | [0.63,1.16] | 0.80 | [0.59,1.08] | 0.81 | [0.63,1.05] | 1.01 | [0.61,1.65] |
| **Dairy product store** | | | |  |  |  |  |  |  |  |
| *1998* (/km^2^) |  |  |  |  |  |  |  |  |  |  |
| 0 (ref) |  |  |  |  |  |  |  |  |  |  |
| >0 | 0.77 | [0.58,1.03] | **0.62^*^** | [0.43,0.91] | 0.93 | [0.63,1.37] | **0.72^*^** | [0.55,0.95] | 1.42 | [0.50,4.06] |
| *1998-2007* |  |  |  |  |  |  |  |  |  |  |
| Decreased | 1.30 | [0.85,1.97] | 1.71 | [0.98,2.98] | 0.97 | [0.51,1.84] | **1.53^*^** | [1.00,2.33] | 0.44 | [0.10,2.00] |
| Constant (ref) |  |  |  |  |  |  |  |  |  |  |
| Increased | 1.07 | [0.85,1.34] | **1.41^*^** | [1.05,1.88] | 0.82 | [0.57,1.17] | 1.05 | [0.81,1.36] | 1.05 | [0.65,1.70] |
| **Health food store** | |  |  |  |  |  |  |  |  |  |
| *1998* (/km^2^) |  |  |  |  |  |  |  |  |  |  |
| 0 (ref) |  |  |  |  |  |  |  |  |  |  |
| >0 | 1.24 | [0.92,1.66] | 1.43 | [0.98,2.08] | 1.09 | [0.69,1.72] | 1.17 | [0.85,1.62] | **2.32^**^** | [1.34,4.01] |
| *1998-2007* |  |  |  |  |  |  |  |  |  |  |
| Decreased | 0.73 | [0.49,1.08] | **0.58^*^** | [0.34,0.99] | 0.89 | [0.49,1.61] | 0.72 | [0.47,1.11] | 0.66 | [0.27,1.63] |
| Constant (ref) |  |  |  |  |  |  |  |  |  |  |
| Increased | 1.03 | [0.82,1.29] | 1.08 | [0.80,1.44] | 0.99 | [0.70,1.40] | 1.00 | [0.76,1.32] | 1.26 | [0.89,1.79] |
| **Candy store** | | |  |  |  |  |  |  |  |  |
| *1998* (/km^2^) |  |  |  |  |  |  |  |  |  |  |
| 0 (ref) |  |  |  |  |  |  |  |  |  |  |
| >0 | 1.55 | [0.68,3.55] | 0.99 | [0.37,2.69] | 2.57 | [0.67,9.82] | 1.46 | [0.63,3.39] | **2.32^**^** | [1.33,4.06] |
| *1998-2007* |  |  |  |  |  |  |  |  |  |  |
| Decreased | 0.79 | [0.31,1.99] | 1.63 | [0.54,4.90] | 0.34 | [0.08,1.44] | 0.81 | [0.31,2.13] | 1.00 | [1.00,1.00] |
| Constant (ref) |  |  |  |  |  |  |  |  |  |  |
| Increased | **1.39^*^** | [1.01,1.93] | 1.00 | [0.65,1.53] | **1.93^**^** | [1.24,3.00] | 1.28 | [0.90,1.80] | **2.10^*^** | [1.09,4.03] |
| **Fruit and vegetable market** | | | |  |  |  |  |  |  |  |
| *1998* (/km^2^) |  |  |  |  |  |  |  |  |  |  |
| 0 (ref) |  |  |  |  |  |  |  |  |  |  |
| >0 | 1.31 | [0.86,1.99] | 1.23 | [0.74,2.05] | 1.62 | [0.85,3.11] | 1.35 | [0.88,2.07] | **0.25^*^** | [0.08,0.83] |
| *1998-2007* |  |  |  |  |  |  |  |  |  |  |
| Decreased | 0.78 | [0.43,1.42] | 1.58 | [0.73,3.40] | **0.37^*^** | [0.15,0.89] | 0.76 | [0.42,1.37] | – | – |
| Constant (ref) |  |  |  |  |  |  |  |  |  |  |
| Increased | 0.72 | [0.51,1.02] | 0.77 | [0.50,1.20] | 0.62 | [0.37,1.02] | 0.75 | [0.54,1.06] | 0.39 | [0.15,1.05] |
| **Meat and fish market** | | | |  |  |  |  |  |  |  |
| *1998* (/km^2^) |  |  |  |  |  |  |  |  |  |  |
| 0 (ref) |  |  |  |  |  |  |  |  |  |  |
| >0 | 0.91 | [0.66,1.25] | 1.19 | [0.79,1.80] | 0.67 | [0.41,1.10] | 0.94 | [0.65,1.35] | 0.66 | [0.32,1.35] |
| *1998-2007* |  |  |  |  |  |  |  |  |  |  |
| Decreased | 0.85 | [0.56,1.31] | 0.60 | [0.35,1.04] | 1.17 | [0.60,2.30] | 0.81 | [0.51,1.31] | – | – |
| Constant (ref) |  |  |  |  |  |  |  |  |  |  |
| Increased | 1.07 | [0.81,1.42] | 0.94 | [0.68,1.30] | 1.22 | [0.84,1.77] | 1.12 | [0.82,1.51] | 0.49 | [0.24,1.03] |
| **Beverage store** | | |  |  |  |  |  |  |  |  |
| *1998* (/km^2^) |  |  |  |  |  |  |  |  |  |  |
| 0 (ref) |  |  |  |  |  |  |  |  |  |  |
| >0 | 0.73 | [0.51,1.05] | 0.94 | [0.56,1.59] | **0.57^*^** | [0.34,0.96] | 0.79 | [0.53,1.19] | 0.53 | [0.21,1.30] |
| *1998-2007* |  |  |  |  |  |  |  |  |  |  |
| Decreased | 1.52 | [0.92,2.51] | 0.84 | [0.42,1.69] | **2.86^**^** | [1.41,5.81] | 1.37 | [0.80,2.33] | – | – |
| Constant (ref) |  |  |  |  |  |  |  |  |  |  |
| Increased | 0.89 | [0.74,1.07] | 0.99 | [0.78,1.25] | 0.83 | [0.62,1.11] | 0.89 | [0.70,1.12] | 0.88 | [0.59,1.32] |

^a^All models were adjusted for age, gender, race/ethnicity, socioeconomic status, parental education, and urbanicity. Boldfaced numbers indicate statistical significance (^*^*p*<0.05, ^**^*p*<0.01, ^***^*p*<0.001).

**Table S5**. Associations (odds ratio and 95% confidence interval) of changes in school neighborhood food environments within 800-m road-network buffer zones during 1998-2007 with child obesity in 2007^a^

| **Food outlet type** | **All**  (n=7,530) | | **Boy**  (n=3,780) | | **Girl**  (n=3,750) | | **Urban**  (n = 5,040) | | **Non-urban**  (n = 2,490) | |
| --- | --- | --- | --- | --- | --- | --- | --- | --- | --- | --- |
| **Supermarket** | | | |  |  |  |  |  |  |  |
| *1998* (/km^2^) |  |  |  |  |  |  |  |  |  |  |
| 0 (ref) |  |  |  |  |  |  |  |  |  |  |
| >0 | 1.10 | [0.88,1.37] | 1.16 | [0.88,1.53] | 1.00 | [0.72,1.39] | 1.13 | [0.88,1.46] | 0.95 | [0.64,1.41] |
| *1998-2007* |  |  |  |  |  |  |  |  |  |  |
| Decreased | 0.96 | [0.73,1.27] | 0.94 | [0.66,1.32] | 0.96 | [0.62,1.48] | 0.94 | [0.68,1.30] | 1.13 | [0.63,2.03] |
| Constant (ref) |  |  |  |  |  |  |  |  |  |  |
| Increased | 1.16 | [0.94,1.42] | 1.10 | [0.83,1.46] | 1.18 | [0.89,1.58] | 1.16 | [0.91,1.47] | 1.28 | [0.86,1.90] |
| **Convenience store** | | | |  |  |  |  |  |  |  |
| *1998* (/km^2^) |  |  |  |  |  |  |  |  |  |  |
| 0 (ref) |  |  |  |  |  |  |  |  |  |  |
| >0 | 0.90 | [0.72,1.13] | **0.70^*^** | [0.53,0.92] | 1.24 | [0.89,1.74] | 0.85 | [0.66,1.08] | 1.13 | [0.62,2.08] |
| *1998-2007* |  |  |  |  |  |  |  |  |  |  |
| Decreased | 1.15 | [0.82,1.61] | **1.58^*^** | [1.07,2.34] | 0.76 | [0.44,1.30] | 1.22 | [0.83,1.78] | 0.71 | [0.34,1.47] |
| Constant (ref) |  |  |  |  |  |  |  |  |  |  |
| Increased | 1.05 | [0.87,1.27] | 1.02 | [0.81,1.30] | 1.16 | [0.87,1.54] | 0.92 | [0.73,1.15] | 1.35 | [0.95,1.92] |
| **Full-service restaurant** | | |  |  |  |  |  |  |  |  |
| *1998* (/km^2^) |  |  |  |  |  |  |  |  |  |  |
| 0 (ref) |  |  |  |  |  |  |  |  |  |  |
| >0 | 0.83 | [0.66,1.03] | 0.75 | [0.55,1.02] | 0.89 | [0.67,1.20] | 0.85 | [0.66,1.10] | 0.92 | [0.56,1.51] |
| *1998-2007* |  |  |  |  |  |  |  |  |  |  |
| Decreased | 1.29 | [0.98,1.70] | **1.45^*^** | [1.01,2.09] | 1.16 | [0.78,1.73] | **1.49^*^** | [1.08,2.07] | 0.88 | [0.50,1.55] |
| Constant (ref) |  |  |  |  |  |  |  |  |  |  |
| Increased | 1.01 | [0.83,1.24] | 0.97 | [0.75,1.25] | 1.10 | [0.82,1.46] | 1.14 | [0.91,1.42] | 0.86 | [0.55,1.36] |
| **Fast-food restaurant** | | |  |  |  |  |  |  |  |  |
| *1998* (/km^2^) |  |  |  |  |  |  |  |  |  |  |
| 0 (ref) |  |  |  |  |  |  |  |  |  |  |
| >0 | 0.97 | [0.76,1.22] | 0.93 | [0.70,1.24] | 1.07 | [0.74,1.55] | 0.86 | [0.67,1.12] | 1.29 | [0.76,2.20] |
| *1998-2007* |  |  |  |  |  |  |  |  |  |  |
| Decreased | 0.78 | [0.54,1.15] | 0.86 | [0.52,1.43] | 0.64 | [0.37,1.10] | 0.84 | [0.56,1.27] | 0.78 | [0.34,1.83] |
| Constant (ref) |  |  |  |  |  |  |  |  |  |  |
| Increased | 1.02 | [0.84,1.24] | 0.97 | [0.75,1.26] | 1.05 | [0.79,1.40] | 1.11 | [0.88,1.40] | 0.72 | [0.49,1.07] |
| **Retail bakery** | | |  |  |  |  |  |  |  |  |
| *1998* (/km^2^) |  |  |  |  |  |  |  |  |  |  |
| 0 (ref) |  |  |  |  |  |  |  |  |  |  |
| >0 | 0.94 | [0.70,1.26] | 0.94 | [0.65,1.35] | 0.93 | [0.59,1.45] | 0.91 | [0.67,1.24] | 0.79 | [0.40,1.58] |
| *1998-2007* |  |  |  |  |  |  |  |  |  |  |
| Decreased | 1.11 | [0.75,1.64] | 1.20 | [0.74,1.95] | 1.12 | [0.62,2.04] | 1.01 | [0.65,1.56] | 2.63 | [0.95,7.24] |
| Constant (ref) |  |  |  |  |  |  |  |  |  |  |
| Increased | 0.94 | [0.72,1.22] | 1.10 | [0.78,1.54] | 0.78 | [0.55,1.10] | 0.93 | [0.71,1.22] | 1.11 | [0.57,2.18] |
| **Dairy product store** | | | |  |  |  |  |  |  |  |
| *1998* (/km^2^) |  |  |  |  |  |  |  |  |  |  |
| 0 (ref) |  |  |  |  |  |  |  |  |  |  |
| >0 | 0.91 | [0.63,1.32] | 0.67 | [0.42,1.09] | 1.27 | [0.79,2.04] | 0.99 | [0.70,1.39] | 0.33 | [0.06,1.86] |
| *1998-2007* |  |  |  |  |  |  |  |  |  |  |
| Decreased | **1.81^*^** | [1.08,3.03] | **2.94^**^** | [1.48,5.85] | 0.87 | [0.45,1.66] | **2.04^**^** | [1.26,3.32] | 0.86 | [0.10,7.42] |
| Constant (ref) |  |  |  |  |  |  |  |  |  |  |
| Increased | 1.02 | [0.77,1.34] | 1.29 | [0.93,1.80] | 0.75 | [0.49,1.17] | 1.01 | [0.75,1.36] | 0.80 | [0.42,1.52] |
| **Health food store** | |  |  |  |  |  |  |  |  |  |
| *1998* (/km^2^) |  |  |  |  |  |  |  |  |  |  |
| 0 (ref) |  |  |  |  |  |  |  |  |  |  |
| >0 | 1.05 | [0.72,1.53] | 1.31 | [0.79,2.18] | 0.75 | [0.44,1.27] | 1.03 | [0.67,1.58] | 1.27 | [0.62,2.60] |
| *1998-2007* |  |  |  |  |  |  |  |  |  |  |
| Decreased | 0.75 | [0.44,1.28] | 0.67 | [0.33,1.35] | 0.96 | [0.49,1.90] | 0.80 | [0.46,1.39] | 0.53 | [0.10,2.91] |
| Constant (ref) |  |  |  |  |  |  |  |  |  |  |
| Increased | 0.92 | [0.70,1.21] | 0.87 | [0.61,1.25] | 1.02 | [0.68,1.52] | 0.99 | [0.73,1.34] | 0.87 | [0.49,1.55] |
| **Candy store** | | |  |  |  |  |  |  |  |  |
| *1998* (/km^2^) |  |  |  |  |  |  |  |  |  |  |
| 0 (ref) |  |  |  |  |  |  |  |  |  |  |
| >0 | 1.50 | [0.68,3.32] | 1.47 | [0.52,4.15] | 1.66 | [0.57,4.87] | 1.45 | [0.67,3.13] | 1.83 | [0.37,9.06] |
| *1998-2007* |  |  |  |  |  |  |  |  |  |  |
| Decreased | 0.71 | [0.29,1.73] | 1.05 | [0.32,3.39] | **0.28^*^** | [0.08,0.94] | 0.80 | [0.33,1.97] | 1.00 | [1.00,1.00] |
| Constant (ref) |  |  |  |  |  |  |  |  |  |  |
| Increased | 1.14 | [0.80,1.65] | 1.24 | [0.77,2.02] | 0.98 | [0.57,1.69] | 1.19 | [0.77,1.82] | 1.25 | [0.67,2.35] |
| **Fruit and vegetable market** | | | |  |  |  |  |  |  |  |
| *1998* (/km^2^) |  |  |  |  |  |  |  |  |  |  |
| 0 (ref) |  |  |  |  |  |  |  |  |  |  |
| >0 | 0.93 | [0.48,1.79] | 0.99 | [0.44,2.27] | 0.89 | [0.31,2.58] | 0.92 | [0.46,1.83] | **0.07^**^** | [0.01,0.43] |
| *1998-2007* |  |  |  |  |  |  |  |  |  |  |
| Decreased | 1.05 | [0.50,2.18] | 0.72 | [0.26,1.99] | 1.39 | [0.48,3.99] | 1.09 | [0.51,2.31] | – | – |
| Constant (ref) |  |  |  |  |  |  |  |  |  |  |
| Increased | 1.16 | [0.77,1.77] | 1.03 | [0.66,1.61] | 1.37 | [0.74,2.56] | 1.33 | [0.91,1.93] | **0.21^*^** | [0.05,0.83] |
| **Meat and fish market** | | | |  |  |  |  |  |  |  |
| *1998* (/km^2^) |  |  |  |  |  |  |  |  |  |  |
| 0 (ref) |  |  |  |  |  |  |  |  |  |  |
| >0 | 0.90 | [0.63,1.28] | 1.00 | [0.63,1.58] | 0.76 | [0.43,1.31] | 0.93 | [0.64,1.36] | 0.53 | [0.18,1.53] |
| *1998-2007* |  |  |  |  |  |  |  |  |  |  |
| Decreased | 0.78 | [0.44,1.40] | 0.57 | [0.28,1.15] | 1.26 | [0.56,2.83] | 0.66 | [0.37,1.21] | – | – |
| Constant (ref) |  |  |  |  |  |  |  |  |  |  |
| Increased | 1.05 | [0.75,1.47] | 1.21 | [0.85,1.73] | 0.87 | [0.51,1.46] | 1.11 | [0.78,1.58] | 0.74 | [0.22,2.49] |
| **Beverage store** | | |  |  |  |  |  |  |  |  |
| *1998* (/km^2^) |  |  |  |  |  |  |  |  |  |  |
| 0 (ref) |  |  |  |  |  |  |  |  |  |  |
| >0 | 0.90 | [0.57,1.44] | 1.06 | [0.63,1.78] | 0.69 | [0.28,1.71] | 0.90 | [0.50,1.63] | 1.07 | [0.46,2.52] |
| *1998-2007* |  |  |  |  |  |  |  |  |  |  |
| Decreased | 1.11 | [0.56,2.21] | 0.88 | [0.41,1.90] | 1.56 | [0.52,4.74] | 1.07 | [0.50,2.30] | – | – |
| Constant (ref) |  |  |  |  |  |  |  |  |  |  |
| Increased | 0.91 | [0.71,1.18] | 1.00 | [0.74,1.36] | 0.80 | [0.54,1.18] | 0.90 | [0.68,1.19] | 0.83 | [0.52,1.34] |

^a^All models were adjusted for age, gender, race/ethnicity, socioeconomic status, parental education, and urbanicity. Boldfaced numbers indicate statistical significance (^*^*p*<0.05, ^**^*p*<0.01, ^***^*p*<0.001).

**Table S6**. Associations (coefficient and standard error) of changes in school neighborhood food environments within ZIP codes during 1998-2007 with child body mass index in 2007^a^

| **Food outlet type** | **All**  (n=7,530) | | **Boy**  (n=3,780) | | **Girl**  (n=3,750) | | **Urban**  (n = 5,040) | | **Non-urban**  (n = 2,490) | |
| --- | --- | --- | --- | --- | --- | --- | --- | --- | --- | --- |
| **Supermarket** | | | |  |  |  |  |  |  |  |
| *1998* (/km^2^) |  |  |  |  |  |  |  |  |  |  |
| 0 (ref) |  |  |  |  |  |  |  |  |  |  |
| >0 | -0.13 | (0.34) | -0.39 | (0.39) | 0.07 | (0.48) | 0.27 | (0.66) | -0.29 | (0.41) |
| *1998-2007* |  |  |  |  |  |  |  |  |  |  |
| Decreased | -0.06 | (0.27) | 0.27 | (0.32) | -0.29 | (0.37) | 0.34 | (0.40) | -0.71 | (0.38) |
| Constant (ref) |  |  |  |  |  |  |  |  |  |  |
| Increased | -0.03 | (0.23) | 0.11 | (0.27) | -0.16 | (0.32) | 0.51 | (0.33) | -0.56 | (0.33) |
| **Convenience store** | | | |  |  |  |  |  |  |  |
| *1998* (/km^2^) |  |  |  |  |  |  |  |  |  |  |
| 0 (ref) |  |  |  |  |  |  |  |  |  |  |
| >0 | 0.26 | (0.24) | **0.66^*^** | (0.27) | -0.17 | (0.34) | 0.43 | (0.34) | 0.11 | (0.34) |
| *1998-2007* |  |  |  |  |  |  |  |  |  |  |
| Decreased | 0.22 | (0.25) | 0.06 | (0.28) | 0.38 | (0.35) | 0.08 | (0.32) | 0.46 | (0.40) |
| Constant (ref) |  |  |  |  |  |  |  |  |  |  |
| Increased | 0.12 | (0.20) | -0.10 | (0.23) | 0.35 | (0.27) | 0.11 | (0.25) | 0.34 | (0.33) |
| **Full-service restaurant** | | |  |  |  |  |  |  |  |  |
| *1998* (/km^2^) |  |  |  |  |  |  |  |  |  |  |
| 0 (ref) |  |  |  |  |  |  |  |  |  |  |
| >0 | -0.33 | (0.38) | -0.29 | (0.42) | -0.38 | (0.54) | -0.91 | (0.76) | -0.22 | (0.44) |
| *1998-2007* |  |  |  |  |  |  |  |  |  |  |
| Decreased | 0.07 | (0.40) | -0.51 | (0.48) | 0.69 | (0.55) | -0.10 | (0.69) | 0.10 | (0.51) |
| Constant (ref) |  |  |  |  |  |  |  |  |  |  |
| Increased | -0.16 | (0.34) | -0.35 | (0.41) | 0.13 | (0.45) | -0.44 | (0.60) | 0.19 | (0.41) |
| **Fast-food restaurant** | | |  |  |  |  |  |  |  |  |
| *1998* (/km^2^) |  |  |  |  |  |  |  |  |  |  |
| 0 (ref) |  |  |  |  |  |  |  |  |  |  |
| >0 | 0.01 | (0.30) | -0.02 | (0.34) | 0.04 | (0.43) | -0.26 | (0.55) | 0.32 | (0.39) |
| *1998-2007* |  |  |  |  |  |  |  |  |  |  |
| Decreased | -0.32 | (0.42) | -0.06 | (0.50) | -0.49 | (0.59) | -0.33 | (0.65) | 0.21 | (0.71) |
| Constant (ref) |  |  |  |  |  |  |  |  |  |  |
| Increased | 0.08 | (0.29) | 0.27 | (0.33) | -0.04 | (0.40) | -0.02 | (0.55) | 0.16 | (0.34) |
| **Retail bakery** | | |  |  |  |  |  |  |  |  |
| *1998* (/km^2^) |  |  |  |  |  |  |  |  |  |  |
| 0 (ref) |  |  |  |  |  |  |  |  |  |  |
| >0 | -0.20 | (0.23) | -0.14 | (0.25) | -0.26 | (0.32) | -0.39 | (0.30) | 0.43 | (0.37) |
| *1998-2007* |  |  |  |  |  |  |  |  |  |  |
| Decreased | -0.18 | (0.25) | 0.02 | (0.29) | -0.30 | (0.36) | -0.06 | (0.31) | -0.64 | (0.45) |
| Constant (ref) |  |  |  |  |  |  |  |  |  |  |
| Increased | 0.03 | (0.20) | 0.29 | (0.23) | -0.18 | (0.28) | 0.23 | (0.24) | -0.43 | (0.38) |
| **Dairy product store** | | | |  |  |  |  |  |  |  |
| *1998* (/km^2^) |  |  |  |  |  |  |  |  |  |  |
| 0 (ref) |  |  |  |  |  |  |  |  |  |  |
| >0 | 0.16 | (0.18) | 0.04 | (0.21) | 0.28 | (0.26) | 0.31 | (0.22) | -0.12 | (0.36) |
| *1998-2007* |  |  |  |  |  |  |  |  |  |  |
| Decreased | -0.36 | (0.28) | 0.00 | (0.33) | -0.77 | (0.40) | -0.41 | (0.32) | -0.84 | (0.68) |
| Constant (ref) |  |  |  |  |  |  |  |  |  |  |
| Increased | 0.09 | (0.18) | 0.20 | (0.20) | -0.06 | (0.25) | 0.11 | (0.22) | -0.19 | (0.32) |
| **Health food store** | |  |  |  |  |  |  |  |  |  |
| *1998* (/km^2^) |  |  |  |  |  |  |  |  |  |  |
| 0 (ref) |  |  |  |  |  |  |  |  |  |  |
| >0 | 0.07 | (0.20) | 0.15 | (0.23) | 0.00 | (0.28) | 0.14 | (0.24) | -0.58 | (0.39) |
| *1998-2007* |  |  |  |  |  |  |  |  |  |  |
| Decreased | **0.49^*^** | (0.24) | -0.15 | (0.28) | **0.94^**^** | (0.35) | 0.30 | (0.28) | **1.50^**^** | (0.58) |
| Constant (ref) |  |  |  |  |  |  |  |  |  |  |
| Increased | 0.14 | (0.17) | 0.14 | (0.20) | 0.05 | (0.25) | 0.15 | (0.21) | -0.10 | (0.30) |
| **Candy store** | | |  |  |  |  |  |  |  |  |
| *1998* (/km^2^) |  |  |  |  |  |  |  |  |  |  |
| 0 (ref) |  |  |  |  |  |  |  |  |  |  |
| >0 | 0.18 | (0.19) | -0.25 | (0.22) | **0.56^*^** | (0.27) | 0.04 | (0.22) | **1.32^**^** | (0.47) |
| *1998-2007* |  |  |  |  |  |  |  |  |  |  |
| Decreased | -0.35 | (0.25) | -0.16 | (0.28) | -0.40 | (0.35) | -0.15 | (0.27) | **-1.58^*^** | (0.71) |
| Constant (ref) |  |  |  |  |  |  |  |  |  |  |
| Increased | -0.10 | (0.17) | -0.36 | (0.19) | 0.20 | (0.23) | -0.15 | (0.19) | 0.25 | (0.32) |
| **Fruit and vegetable market** | | | |  |  |  |  |  |  |  |
| *1998* (/km^2^) |  |  |  |  |  |  |  |  |  |  |
| 0 (ref) |  |  |  |  |  |  |  |  |  |  |
| >0 | 0.16 | (0.19) | 0.30 | (0.21) | 0.02 | (0.26) | -0.03 | (0.22) | **0.73^*^** | (0.36) |
| *1998-2007* |  |  |  |  |  |  |  |  |  |  |
| Decreased | 0.01 | (0.29) | 0.08 | (0.33) | -0.18 | (0.43) | 0.23 | (0.32) | -1.07 | (0.78) |
| Constant (ref) |  |  |  |  |  |  |  |  |  |  |
| Increased | -0.24 | (0.16) | -0.21 | (0.19) | -0.26 | (0.23) | -0.27 | (0.19) | -0.45 | (0.32) |
| **Meat and fish market** | | | |  |  |  |  |  |  |  |
| *1998* (/km^2^) |  |  |  |  |  |  |  |  |  |  |
| 0 (ref) |  |  |  |  |  |  |  |  |  |  |
| >0 | 0.20 | (0.19) | 0.03 | (0.21) | 0.40 | (0.27) | 0.11 | (0.21) | 0.53 | (0.48) |
| *1998-2007* |  |  |  |  |  |  |  |  |  |  |
| Decreased | -0.37 | (0.24) | 0.13 | (0.28) | **-0.74^*^** | (0.35) | -0.24 | (0.27) | -0.78 | (0.70) |
| Constant (ref) |  |  |  |  |  |  |  |  |  |  |
| Increased | -0.06 | (0.16) | -0.03 | (0.19) | -0.07 | (0.23) | -0.01 | (0.19) | -0.23 | (0.32) |
| **Beverage store** | | |  |  |  |  |  |  |  |  |
| *1998* (/km^2^) |  |  |  |  |  |  |  |  |  |  |
| 0 (ref) |  |  |  |  |  |  |  |  |  |  |
| >0 | -0.34 | (0.18) | -0.05 | (0.20) | **-0.60^*^** | (0.25) | -0.22 | (0.20) | -0.51 | (0.41) |
| *1998-2007* |  |  |  |  |  |  |  |  |  |  |
| Decreased | 0.61 | (0.34) | **0.83^*^** | (0.41) | 0.30 | (0.50) | 0.62 | (0.37) | 0.21 | (1.00) |
| Constant (ref) |  |  |  |  |  |  |  |  |  |  |
| Increased | -0.21 | (0.18) | -0.21 | (0.21) | -0.21 | (0.25) | -0.28 | (0.22) | -0.03 | (0.33) |

^a^All models were adjusted for age, gender, race/ethnicity, socioeconomic status, parental education, and urbanicity. Boldfaced numbers indicate statistical significance (^*^*p*<0.05, ^**^*p*<0.01, ^***^*p*<0.001).

**Table S7**. Associations (odds ratio and 95% confidence interval) of changes in school neighborhood food environments within ZIP codes during 1998-2007 with child overweight and obesity in 2007^a^

| **Food outlet type** | **All**  (n=7,530) | | **Boy**  (n=3,780) | | **Girl**  (n=3,750) | | **Urban**  (n = 5,040) | | **Non-urban**  (n = 2,490) | |
| --- | --- | --- | --- | --- | --- | --- | --- | --- | --- | --- |
| **Supermarket** | | | |  |  |  |  |  |  |  |
| *1998* (/km^2^) |  |  |  |  |  |  |  |  |  |  |
| 0 (ref) |  |  |  |  |  |  |  |  |  |  |
| >0 | 1.07 | [0.78,1.45] | 1.02 | [0.68,1.54] | 1.06 | [0.71,1.58] | 1.18 | [0.73,1.93] | 0.94 | [0.66,1.35] |
| *1998-2007* |  |  |  |  |  |  |  |  |  |  |
| Decreased | 0.96 | [0.76,1.20] | 0.95 | [0.68,1.31] | 0.97 | [0.71,1.33] | 0.93 | [0.66,1.31] | 0.95 | [0.69,1.31] |
| Constant (ref) |  |  |  |  |  |  |  |  |  |  |
| Increased | 0.97 | [0.80,1.18] | 0.96 | [0.74,1.25] | 0.98 | [0.74,1.29] | 1.02 | [0.76,1.36] | 0.92 | [0.70,1.21] |
| **Convenience store** | | | |  |  |  |  |  |  |  |
| *1998* (/km^2^) |  |  |  |  |  |  |  |  |  |  |
| 0 (ref) |  |  |  |  |  |  |  |  |  |  |
| >0 | 1.00 | [0.83,1.20] | 1.17 | [0.91,1.49] | 0.86 | [0.65,1.12] | 1.15 | [0.88,1.50] | 0.84 | [0.66,1.08] |
| *1998-2007* |  |  |  |  |  |  |  |  |  |  |
| Decreased | 1.13 | [0.92,1.37] | 1.13 | [0.87,1.46] | 1.11 | [0.83,1.48] | 1.05 | [0.81,1.36] | 1.27 | [0.93,1.75] |
| Constant (ref) |  |  |  |  |  |  |  |  |  |  |
| Increased | 1.17 | [1.00,1.37] | 1.21 | [0.97,1.51] | 1.14 | [0.92,1.41] | 1.13 | [0.93,1.38] | 1.23 | [0.93,1.64] |
| **Full-service restaurant** | | |  |  |  |  |  |  |  |  |
| *1998* (/km^2^) |  |  |  |  |  |  |  |  |  |  |
| 0 (ref) |  |  |  |  |  |  |  |  |  |  |
| >0 | 1.35 | [0.99,1.85] | 1.21 | [0.81,1.82] | **1.61^*^** | [1.05,2.47] | 1.22 | [0.66,2.26] | **1.53^*^** | [1.05,2.24] |
| *1998-2007* |  |  |  |  |  |  |  |  |  |  |
| Decreased | 0.81 | [0.60,1.08] | 0.64 | [0.41,1.01] | 1.00 | [0.69,1.47] | 0.81 | [0.49,1.33] | 0.78 | [0.52,1.15] |
| Constant (ref) |  |  |  |  |  |  |  |  |  |  |
| Increased | 0.81 | [0.63,1.04] | 0.72 | [0.48,1.08] | 0.90 | [0.67,1.21] | 0.72 | [0.47,1.10] | 0.91 | [0.65,1.27] |
| **Fast-food restaurant** | | |  |  |  |  |  |  |  |  |
| *1998* (/km^2^) |  |  |  |  |  |  |  |  |  |  |
| 0 (ref) |  |  |  |  |  |  |  |  |  |  |
| >0 | 0.96 | [0.76,1.22] | 0.87 | [0.66,1.17] | 1.03 | [0.73,1.44] | 0.96 | [0.60,1.53] | 0.99 | [0.71,1.37] |
| *1998-2007* |  |  |  |  |  |  |  |  |  |  |
| Decreased | 0.97 | [0.68,1.38] | 1.24 | [0.75,2.06] | 0.76 | [0.48,1.20] | 0.76 | [0.44,1.32] | 1.18 | [0.55,2.53] |
| Constant (ref) |  |  |  |  |  |  |  |  |  |  |
| Increased | 0.98 | [0.77,1.24] | 0.98 | [0.69,1.38] | 0.99 | [0.73,1.34] | 0.84 | [0.53,1.33] | 1.02 | [0.76,1.37] |
| **Retail bakery** | | |  |  |  |  |  |  |  |  |
| *1998* (/km^2^) |  |  |  |  |  |  |  |  |  |  |
| 0 (ref) |  |  |  |  |  |  |  |  |  |  |
| >0 | 0.84 | [0.71,1.00] | 0.97 | [0.76,1.24] | **0.72^**^** | [0.56,0.91] | 0.82 | [0.65,1.04] | 0.89 | [0.66,1.20] |
| *1998-2007* |  |  |  |  |  |  |  |  |  |  |
| Decreased | 1.16 | [0.94,1.45] | 1.18 | [0.88,1.59] | 1.12 | [0.81,1.54] | 1.27 | [0.97,1.68] | 1.02 | [0.70,1.48] |
| Constant (ref) |  |  |  |  |  |  |  |  |  |  |
| Increased | 1.04 | [0.87,1.24] | 1.19 | [0.94,1.50] | 0.90 | [0.70,1.14] | 1.15 | [0.93,1.43] | 0.83 | [0.57,1.20] |
| **Dairy product store** | | | |  |  |  |  |  |  |  |
| *1998* (/km^2^) |  |  |  |  |  |  |  |  |  |  |
| 0 (ref) |  |  |  |  |  |  |  |  |  |  |
| >0 | 1.05 | [0.89,1.24] | 0.86 | [0.70,1.07] | **1.35^*^** | [1.07,1.71] | 1.04 | [0.85,1.28] | 1.09 | [0.82,1.44] |
| *1998-2007* |  |  |  |  |  |  |  |  |  |  |
| Decreased | 0.89 | [0.71,1.11] | 1.30 | [0.93,1.80] | **0.60^**^** | [0.43,0.84] | 0.90 | [0.70,1.16] | 0.87 | [0.52,1.46] |
| Constant (ref) |  |  |  |  |  |  |  |  |  |  |
| Increased | 0.89 | [0.77,1.04] | 1.03 | [0.84,1.26] | **0.77^*^** | [0.62,0.96] | 0.88 | [0.72,1.07] | 0.93 | [0.69,1.24] |
| **Health food store** | |  |  |  |  |  |  |  |  |  |
| *1998* (/km^2^) |  |  |  |  |  |  |  |  |  |  |
| 0 (ref) |  |  |  |  |  |  |  |  |  |  |
| >0 | 1.07 | [0.90,1.27] | 1.06 | [0.84,1.34] | 1.06 | [0.83,1.36] | 1.14 | [0.92,1.42] | 0.83 | [0.61,1.13] |
| *1998-2007* |  |  |  |  |  |  |  |  |  |  |
| Decreased | 1.02 | [0.82,1.26] | 0.88 | [0.67,1.16] | 1.16 | [0.85,1.60] | 1.02 | [0.79,1.31] | 1.17 | [0.72,1.91] |
| Constant (ref) |  |  |  |  |  |  |  |  |  |  |
| Increased | 1.10 | [0.95,1.28] | 1.15 | [0.95,1.41] | 1.04 | [0.85,1.29] | 1.13 | [0.94,1.36] | 1.06 | [0.80,1.41] |
| **Candy store** | | |  |  |  |  |  |  |  |  |
| *1998* (/km^2^) |  |  |  |  |  |  |  |  |  |  |
| 0 (ref) |  |  |  |  |  |  |  |  |  |  |
| >0 | 0.93 | [0.78,1.10] | 0.81 | [0.64,1.02] | 1.07 | [0.82,1.38] | 0.92 | [0.76,1.13] | 0.93 | [0.64,1.36] |
| *1998-2007* |  |  |  |  |  |  |  |  |  |  |
| Decreased | 1.06 | [0.85,1.31] | 0.90 | [0.68,1.21] | 1.30 | [0.94,1.78] | 1.04 | [0.82,1.32] | 1.35 | [0.71,2.58] |
| Constant (ref) |  |  |  |  |  |  |  |  |  |  |
| Increased | 0.98 | [0.84,1.14] | **0.80^*^** | [0.66,0.98] | 1.23 | [0.99,1.52] | 0.95 | [0.79,1.15] | 1.13 | [0.84,1.51] |
| **Fruit and vegetable market** | | | |  |  |  |  |  |  |  |
| *1998* (/km^2^) |  |  |  |  |  |  |  |  |  |  |
| 0 (ref) |  |  |  |  |  |  |  |  |  |  |
| >0 | 1.08 | [0.92,1.27] | 1.22 | [0.99,1.51] | 0.95 | [0.75,1.20] | 1.08 | [0.89,1.31] | 1.12 | [0.81,1.55] |
| *1998-2007* |  |  |  |  |  |  |  |  |  |  |
| Decreased | 0.92 | [0.71,1.20] | 0.93 | [0.67,1.30] | 0.89 | [0.59,1.32] | 0.95 | [0.70,1.30] | 0.61 | [0.33,1.12] |
| Constant (ref) |  |  |  |  |  |  |  |  |  |  |
| Increased | 0.87 | [0.76,1.01] | 0.90 | [0.74,1.08] | 0.88 | [0.71,1.09] | 0.91 | [0.77,1.07] | 0.74 | [0.53,1.04] |
| **Meat and fish market** | | | |  |  |  |  |  |  |  |
| *1998* (/km^2^) |  |  |  |  |  |  |  |  |  |  |
| 0 (ref) |  |  |  |  |  |  |  |  |  |  |
| >0 | 0.89 | [0.76,1.04] | **0.80^*^** | [0.65,1.00] | 0.98 | [0.77,1.24] | 0.87 | [0.72,1.05] | 0.90 | [0.66,1.23] |
| *1998-2007* |  |  |  |  |  |  |  |  |  |  |
| Decreased | 1.10 | [0.89,1.36] | **1.44^**^** | [1.10,1.89] | 0.87 | [0.61,1.23] | 1.10 | [0.87,1.39] | 1.28 | [0.69,2.38] |
| Constant (ref) |  |  |  |  |  |  |  |  |  |  |
| Increased | 1.06 | [0.92,1.22] | 1.14 | [0.94,1.38] | 1.01 | [0.83,1.24] | 1.08 | [0.91,1.29] | 1.02 | [0.80,1.31] |
| **Beverage store** | | |  |  |  |  |  |  |  |  |
| *1998* (/km^2^) |  |  |  |  |  |  |  |  |  |  |
| 0 (ref) |  |  |  |  |  |  |  |  |  |  |
| >0 | 0.98 | [0.84,1.14] | 1.13 | [0.91,1.40] | 0.86 | [0.70,1.06] | 0.94 | [0.79,1.12] | 1.14 | [0.80,1.63] |
| *1998-2007* |  |  |  |  |  |  |  |  |  |  |
| Decreased | 1.12 | [0.84,1.49] | 1.24 | [0.85,1.83] | 0.91 | [0.57,1.45] | 1.15 | [0.83,1.59] | 0.87 | [0.47,1.61] |
| Constant (ref) |  |  |  |  |  |  |  |  |  |  |
| Increased | 1.00 | [0.85,1.18] | 1.07 | [0.88,1.32] | 0.91 | [0.72,1.14] | 0.96 | [0.78,1.18] | 1.16 | [0.89,1.52] |

^a^All models were adjusted for age, gender, race/ethnicity, socioeconomic status, parental education, and urbanicity. Boldfaced numbers indicate statistical significance (^*^*p*<0.05, ^**^*p*<0.01, ^***^*p*<0.001).

**Table S8**. Associations (odds ratio and 95% confidence interval) of changes in school neighborhood food environments within ZIP codes during 1998-2007 with child obesity in 2007^a^

| **Food outlet type** | **All**  (n=7,530) | | **Boy**  (n=3,780) | | **Girl**  (n=3,750) | | **Urban**  (n = 5,040) | | **Non-urban**  (n = 2,490) | |
| --- | --- | --- | --- | --- | --- | --- | --- | --- | --- | --- |
| **Supermarket** | | | |  |  |  |  |  |  |  |
| *1998* (/km^2^) |  |  |  |  |  |  |  |  |  |  |
| 0 (ref) |  |  |  |  |  |  |  |  |  |  |
| >0 | 1.02 | [0.71,1.46] | 0.96 | [0.60,1.53] | 1.03 | [0.64,1.66] | 1.19 | [0.60,2.34] | 0.96 | [0.61,1.52] |
| *1998-2007* |  |  |  |  |  |  |  |  |  |  |
| Decreased | 1.27 | [0.93,1.73] | 1.45 | [0.99,2.14] | 1.14 | [0.74,1.76] | 1.50 | [0.96,2.36] | 0.95 | [0.60,1.51] |
| Constant (ref) |  |  |  |  |  |  |  |  |  |  |
| Increased | 1.17 | [0.90,1.51] | 1.26 | [0.90,1.75] | 1.09 | [0.75,1.56] | 1.46 | [1.00,2.15] | 0.92 | [0.63,1.34] |
| **Convenience store** | | | |  |  |  |  |  |  |  |
| *1998* (/km^2^) |  |  |  |  |  |  |  |  |  |  |
| 0 (ref) |  |  |  |  |  |  |  |  |  |  |
| >0 | 1.21 | [0.94,1.55] | 1.33 | [0.96,1.86] | 1.07 | [0.74,1.55] | 1.17 | [0.81,1.68] | 1.21 | [0.84,1.76] |
| *1998-2007* |  |  |  |  |  |  |  |  |  |  |
| Decreased | 1.19 | [0.92,1.55] | 1.13 | [0.81,1.59] | 1.24 | [0.83,1.84] | 1.16 | [0.84,1.60] | 1.20 | [0.79,1.81] |
| Constant (ref) |  |  |  |  |  |  |  |  |  |  |
| Increased | 1.18 | [0.95,1.47] | 1.12 | [0.84,1.49] | 1.23 | [0.88,1.71] | 1.08 | [0.84,1.38] | 1.49 | [0.99,2.24] |
| **Full-service restaurant** | | |  |  |  |  |  |  |  |  |
| *1998* (/km^2^) |  |  |  |  |  |  |  |  |  |  |
| 0 (ref) |  |  |  |  |  |  |  |  |  |  |
| >0 | 0.99 | [0.68,1.44] | 1.12 | [0.69,1.81] | 1.00 | [0.60,1.67] | 0.60 | [0.27,1.33] | 1.27 | [0.80,2.03] |
| *1998-2007* |  |  |  |  |  |  |  |  |  |  |
| Decreased | 1.02 | [0.67,1.56] | 0.97 | [0.56,1.67] | 0.96 | [0.53,1.74] | 0.90 | [0.47,1.73] | 0.84 | [0.46,1.52] |
| Constant (ref) |  |  |  |  |  |  |  |  |  |  |
| Increased | 0.96 | [0.67,1.36] | 0.81 | [0.51,1.30] | 1.10 | [0.68,1.79] | 0.69 | [0.40,1.18] | 1.22 | [0.75,1.96] |
| **Fast-food restaurant** | | |  |  |  |  |  |  |  |  |
| *1998* (/km^2^) |  |  |  |  |  |  |  |  |  |  |
| 0 (ref) |  |  |  |  |  |  |  |  |  |  |
| >0 | 0.91 | [0.68,1.22] | 0.80 | [0.57,1.12] | 1.00 | [0.64,1.56] | 1.03 | [0.60,1.76] | 0.92 | [0.60,1.41] |
| *1998-2007* |  |  |  |  |  |  |  |  |  |  |
| Decreased | 1.03 | [0.66,1.60] | 0.97 | [0.54,1.73] | 1.17 | [0.66,2.07] | 1.31 | [0.66,2.60] | 1.11 | [0.48,2.55] |
| Constant (ref) |  |  |  |  |  |  |  |  |  |  |
| Increased | 0.92 | [0.70,1.20] | 1.02 | [0.71,1.46] | 0.86 | [0.58,1.26] | 1.21 | [0.70,2.12] | 0.88 | [0.61,1.27] |
| **Retail bakery** | | |  |  |  |  |  |  |  |  |
| *1998* (/km^2^) |  |  |  |  |  |  |  |  |  |  |
| 0 (ref) |  |  |  |  |  |  |  |  |  |  |
| >0 | 0.88 | [0.70,1.12] | 0.92 | [0.70,1.22] | 0.81 | [0.58,1.15] | 0.79 | [0.59,1.05] | 1.14 | [0.74,1.78] |
| *1998-2007* |  |  |  |  |  |  |  |  |  |  |
| Decreased | 1.00 | [0.76,1.31] | 1.06 | [0.76,1.48] | 0.94 | [0.61,1.43] | 0.92 | [0.67,1.27] | 1.00 | [0.57,1.78] |
| Constant (ref) |  |  |  |  |  |  |  |  |  |  |
| Increased | 1.05 | [0.85,1.31] | 1.28 | [0.99,1.65] | 0.83 | [0.61,1.13] | 1.07 | [0.83,1.38] | 0.84 | [0.53,1.34] |
| **Dairy product store** | | | |  |  |  |  |  |  |  |
| *1998* (/km^2^) |  |  |  |  |  |  |  |  |  |  |
| 0 (ref) |  |  |  |  |  |  |  |  |  |  |
| >0 | 1.10 | [0.89,1.35] | 1.03 | [0.81,1.31] | 1.18 | [0.87,1.61] | 1.16 | [0.91,1.49] | 1.07 | [0.70,1.64] |
| *1998-2007* |  |  |  |  |  |  |  |  |  |  |
| Decreased | 0.91 | [0.66,1.26] | 1.07 | [0.69,1.65] | 0.73 | [0.47,1.14] | 0.86 | [0.59,1.25] | 0.95 | [0.47,1.90] |
| Constant (ref) |  |  |  |  |  |  |  |  |  |  |
| Increased | 1.02 | [0.85,1.24] | 1.01 | [0.79,1.29] | 1.06 | [0.80,1.39] | 1.12 | [0.87,1.43] | 0.80 | [0.55,1.15] |
| **Health food store** | |  |  |  |  |  |  |  |  |  |
| *1998* (/km^2^) |  |  |  |  |  |  |  |  |  |  |
| 0 (ref) |  |  |  |  |  |  |  |  |  |  |
| >0 | 0.93 | [0.75,1.16] | 1.00 | [0.76,1.30] | 0.82 | [0.59,1.14] | 0.97 | [0.74,1.27] | 0.68 | [0.46,1.02] |
| *1998-2007* |  |  |  |  |  |  |  |  |  |  |
| Decreased | 1.01 | [0.78,1.31] | 0.75 | [0.54,1.05] | 1.46 | [0.98,2.17] | 0.92 | [0.69,1.23] | 1.74 | [0.96,3.15] |
| Constant (ref) |  |  |  |  |  |  |  |  |  |  |
| Increased | 1.06 | [0.87,1.29] | 1.03 | [0.82,1.31] | 1.08 | [0.81,1.42] | 1.06 | [0.84,1.34] | 0.98 | [0.69,1.40] |
| **Candy store** | | |  |  |  |  |  |  |  |  |
| *1998* (/km^2^) |  |  |  |  |  |  |  |  |  |  |
| 0 (ref) |  |  |  |  |  |  |  |  |  |  |
| >0 | 0.85 | [0.68,1.06] | **0.73^*^** | [0.55,0.97] | 1.02 | [0.74,1.39] | 0.82 | [0.64,1.06] | 1.10 | [0.66,1.83] |
| *1998-2007* |  |  |  |  |  |  |  |  |  |  |
| Decreased | 1.06 | [0.80,1.40] | 1.18 | [0.83,1.67] | 0.98 | [0.65,1.48] | 1.08 | [0.80,1.45] | 1.01 | [0.46,2.23] |
| Constant (ref) |  |  |  |  |  |  |  |  |  |  |
| Increased | 1.02 | [0.85,1.23] | 0.98 | [0.78,1.23] | 1.09 | [0.82,1.44] | 0.93 | [0.75,1.16] | 1.29 | [0.86,1.93] |
| **Fruit and vegetable market** | | | |  |  |  |  |  |  |  |
| *1998* (/km^2^) |  |  |  |  |  |  |  |  |  |  |
| 0 (ref) |  |  |  |  |  |  |  |  |  |  |
| >0 | 1.01 | [0.83,1.22] | 1.12 | [0.87,1.43] | 0.88 | [0.65,1.18] | 0.97 | [0.78,1.21] | 1.14 | [0.78,1.66] |
| *1998-2007* |  |  |  |  |  |  |  |  |  |  |
| Decreased | 1.04 | [0.76,1.41] | 0.94 | [0.64,1.38] | 1.15 | [0.70,1.89] | 1.18 | [0.85,1.65] | **0.38^**^** | [0.20,0.73] |
| Constant (ref) |  |  |  |  |  |  |  |  |  |  |
| Increased | 0.90 | [0.75,1.08] | 0.86 | [0.69,1.08] | 0.96 | [0.74,1.26] | 0.94 | [0.77,1.15] | 0.70 | [0.47,1.05] |
| **Meat and fish market** | | | |  |  |  |  |  |  |  |
| *1998* (/km^2^) |  |  |  |  |  |  |  |  |  |  |
| 0 (ref) |  |  |  |  |  |  |  |  |  |  |
| >0 | 1.04 | [0.85,1.26] | 0.87 | [0.68,1.12] | 1.30 | [0.97,1.75] | 1.04 | [0.83,1.30] | 1.03 | [0.62,1.71] |
| *1998-2007* |  |  |  |  |  |  |  |  |  |  |
| Decreased | 0.95 | [0.73,1.22] | 1.37 | [0.99,1.90] | **0.64^*^** | [0.42,0.97] | 0.99 | [0.74,1.33] | 1.01 | [0.54,1.88] |
| Constant (ref) |  |  |  |  |  |  |  |  |  |  |
| Increased | 1.03 | [0.86,1.23] | 1.17 | [0.93,1.48] | 0.88 | [0.68,1.15] | 1.06 | [0.86,1.30] | 1.06 | [0.73,1.54] |
| **Beverage store** | | |  |  |  |  |  |  |  |  |
| *1998* (/km^2^) |  |  |  |  |  |  |  |  |  |  |
| 0 (ref) |  |  |  |  |  |  |  |  |  |  |
| >0 | 0.96 | [0.80,1.15] | 1.03 | [0.81,1.30] | 0.90 | [0.69,1.17] | 1.03 | [0.85,1.25] | 0.82 | [0.52,1.31] |
| *1998-2007* |  |  |  |  |  |  |  |  |  |  |
| Decreased | 1.20 | [0.84,1.73] | **1.70^*^** | [1.04,2.77] | 0.74 | [0.43,1.27] | 1.28 | [0.86,1.92] | 0.90 | [0.45,1.77] |
| Constant (ref) |  |  |  |  |  |  |  |  |  |  |
| Increased | 0.99 | [0.81,1.21] | 1.12 | [0.87,1.43] | 0.85 | [0.65,1.11] | 1.04 | [0.82,1.32] | 0.95 | [0.64,1.41] |

^a^All models were adjusted for age, gender, race/ethnicity, socioeconomic status, parental education, and urbanicity. Boldfaced numbers indicate statistical significance (^*^*p*<0.05, ^**^*p*<0.01, ^***^*p*<0.001).
